# Gut bacterial aromatic amine production: aromatic amino acid decarboxylase and its effects on peripheral serotonin production

**DOI:** 10.1080/19490976.2022.2128605

**Published:** 2022-10-10

**Authors:** Yuta Sugiyama, Yumiko Mori, Misaki Nara, Yusuke Kotani, Emiko Nagai, Hiroki Kawada, Mayu Kitamura, Rika Hirano, Hiromi Shimokawa, Akira Nakagawa, Hiromichi Minami, Aina Gotoh, Mikiyasu Sakanaka, Noriho Iida, Takashi Koyanagi, Takane Katayama, Shigefumi Okamoto, Shin Kurihara

**Affiliations:** a Faculty of Bioresources and Environmental Sciences, Ishikawa Prefectural University, Nonoichi, 921-8836, Japan; b Gunma University Center for Food Science and Wellness, Gunma University, Maebashi, Japan; c Department of Clinical Laboratory Sciences, Faculty of Health Sciences, Institute of Medical, Pharmaceutical, and Health Sciences, Kanazawa University, Kanazawa, Japan; d Department of Biotechnology, Graduate School of Agricultural and Life Sciences, the University of Tokyo, Bunkyo-ku, Japan; e Faculty of Biology-Oriented Science and Technology, Kindai University, Kinokawa, Japan; f Graduate School of Biostudies, Kyoto University, Kyoto, Japan; g Department of Gastroenterology, Graduate School of Medicine, Kanazawa University, Kanazawa, Japan; h Advanced Health Care Science Research Unit, Innovative Integrated Bio-Research Core, Institute for Frontier Science Initiative, Kanazawa University, Kanazawa, Japan

**Keywords:** Aromatic amine, aromatic amino acid, peripheral serotonin, *Enterococcus faecalis*, gut microbiota, aromatic amino acid decarboxylase, aromatic amino acid decarboxylase inhibitor

## Abstract

Colonic luminal aromatic amines have been historically considered to be derived from dietary source, especially fermented foods; however, recent studies indicate that the gut microbiota serves as an alternative source of these amines. Herein, we show that five prominent genera of Firmicutes *(Blautia, Clostridium, Enterococcus, Ruminococcus*, and *Tyzzerella*) have the ability to abundantly produce aromatic amines through the action of aromatic amino acid decarboxylase (AADC). *In vitro* cultivation of human fecal samples revealed that a significant positive correlation between *aadc* copy number of *Ruminococcus gnavus* and phenylethylamine (PEA) production. Furthermore, using genetically engineered *Enterococcus faecalis*-colonized BALB/cCrSlc mouse model, we showed that the gut bacterial *aadc* stimulates the production of colonic serotonin, which is reportedly involved in osteoporosis and irritable bowel syndrome. Finally, we showed that human AADC inhibitors carbidopa and benserazide inhibit PEA production in *En. faecalis*.

## Introduction

The effects of gut bacterial metabolites on host health have been demonstrated in rodent models. For example, acetic acid protects the host against pathogen infection^1^, butyric acid induces the colonic regulatory T cell differentiation,^2^ deoxycholic acid increases liver cancer incidence,^3^ lithocholic acid ameliorates inflammation in colitis,^4^ and polyamines extend host longevity,^5,6^ and maintain mucosal homeostasis.^7^ The importance of gut bacterial metabolites is also recognized in humans has also been reported, e.g., de novo biosynthesis of vitamin K (menaquinones), which humans cannot biosynthesize, in gut.^8^ Moreover, polyamines produced by gut bacteria contribute to atherosclerosis prevention.^9^ Considering these findings, the regulation of gut bacterial metabolite production in the human is crucial for the maintenance of nutritional homeostasis and diseases prevention in humans. The molecular mechanism of gut bacterial metabolites production and their physiological function in the host have been gradually elucidated. For example, aromatic lactic acid produced by aromatic lactate dehydrogenase of *Bifidobacterium* affects the host immune system,^10^ and isoallolithocholic acid produced by 5α-reductase and 3β-hydroxysteroid dehydrogenase of *Parabacteroides merdae* St3 through shows bactericidal activity against gram-positive pathogens.^11^ However, our knowledge about the sources of gut bacterial metabolites and their production mechanism and physiological effects on host remains fragmentary.

Aromatic amines are among the compounds detected in the colon;^12,13^ tryptamine, tyramine, and phenylethylamine (PEA) are typically known as the trace amines that can affect neurotransmission even in small amounts.^14,15^ Diet and gut microbiota have been identified as sources of colonic luminal aromatic amines. Aromatic amines are found in fermented foods,^16^ nuts, and citrus fruits^17^ as well as in roasted coffee and cacao, where they are generated by Maillard- and Strecker reactions.^17–19^ Several previous studies have reported that some gut bacteria possess aromatic amine-producing capabilities^20^ and contribute to colonic aromatic amine level using germ-free animal models such as rats, chickens, and mice.^21–24^ Previous *in vitro* studies have revealed that gut bacteria produce aromatic amines from aromatic amino acids through decarboxylation catalyzed by aromatic amino acid decarboxylase (AADC).^23,25^ The kinetic parameters of AADC from several bacterial species, including gut bacteria (such as *Enterococcus faecalis, Clostridium sporogenes*, and *Ruminococcus gnavus*), have been determined using purified recombinant enzymes.^25,26^ Almost all characterized AADC has shown relatively broad substrate specificity,^25,27^ catalyzing the decarboxylation of not only proteinogenic aromatic amino acids (Phe, Tyr, and Trp), but also non-proteinogenic aromatic amino acids such as 3,4-dihydroxyphenylalanine (L-DOPA)^28–31^ and 5-hydroxytryptophan.^27^ Most previous studies on aromatic amino acid decarboxylation and aromatic amine production during the cultivation were focused on aromatic amine production from a non-proteinogenic aromatic amino acid L-DOPA.^29,32^ Gut bacteria produce biogenic amines, including aromatic amines, from proteinogenic amino acids abundant in the diet,^33,34^ such as tryptamine synthesis through the decarboxylation of tryptophan by gut bacteria.^25^ However, few studies have elucidated at the genetic level how gut bacteria contribute to the production of trace amines from proteinogenic aromatic amino acids in the colon, and further research is needed to elucidate it.

PEA has been shown to induce efflux of neurotransmitters (dopamine, norepinephrine, and serotonin) and inhibit uptake of these neurotransmitters in human cell lines and in brain synaptosomes from mouse, juvenile rhesus, and tamarin.^35^ PEA activates polymorphonuclear leukocytes and induces allergic reactions.^36^ These physiological effects of PEA are mediated by trace amine-associated receptor 1 (TAAR1) activation.^35,36^ Because TAAR1 is also expressed in the colonic epithelium,^37^ PEA from gut bacteria is expected to exert physiological functions through TAAR1.^38^ However, no studies have been conducted to investigate this possibility.

Serotonin (5-hydroxytryptamine) is a monoamine neurotransmitter distributed in the central and peripheral nervous systems, and plays different roles depending on location. Serotonin in the central nervous system is a neurotransmitter in the brain and affects sleep^39^ and appetite,^40^ whereas peripheral serotonin is a regulatory factor in different organs, regulating bone development,^41,42^ immune response,^43^ and brown adipose tissue thermogenesis.^44^ Peripheral serotonin, which accounts for 90% of serotonin in the body, is produced by enterochromaffin (EC) cells in the gastrointestinal tract.^45^ Bhattarai *et al*. demonstrated that *Bacteroides thetaiotaomicron* heterologously expressing *aadc* of *R. gnavus* produced tryptamine in the mouse gut, and the produced tryptamine increased anion and fluid secretion in the proximal colon via Serotonin receptor-4, one of the G protein-coupled receptors (GPCRs).^23^ Gut bacteria modulate gastrointestinal motility and platelet function by promoting peripheral serotonin production from EC cells, mediated by tyramine and other gut bacterial metabolites.^24^ Therefore, aromatic amines produced by gut bacteria have a significant effect on host physiology, by way of serotonin^24^ or serotonin signaling pathways.^23,46^ However, the serotonin-mediated relationship between the host physiology and PEA, another natural aromatic amine, remains to be studied.

In this study, we identified five species of PEA-producing gut bacteria among 32 species of dominant human gut bacteria and verified that PEA production depends on *aadc*. The effects of *aadc* on colonic luminal aromatic amine and colonic serotonin production in the host were evaluated using mouse model. In addition, the PEA production by *En. faecalis* was successfully inhibited using established inhibitors.

## Results

### Discovery of the PEA-producing bacteria in the most predominant species in the human indigenous gut microbiota

**Figure 1.**
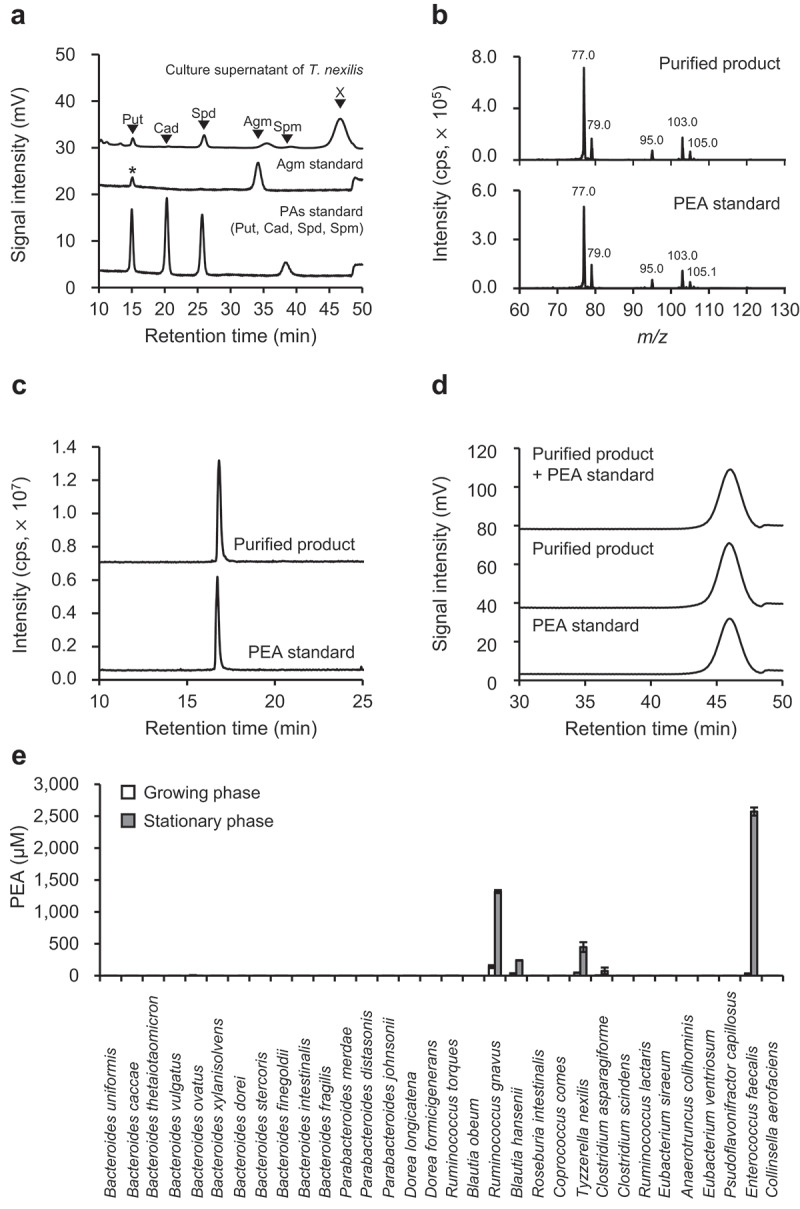
Identification of PEA and PEA-producing gut bacteria. (a) Unidentified biogenic amine (x) observed in *B. hansenii, C. asparagiforme, T. nexilis, En. faecalis*, and *R. gnavus*. The upper panel shows the HPLC chromatogram of culture supernatant of *T. nexilis* as representative data, and the lower and middle panels are chromatograms of polyamines (PAs) standard (Put, putrescine; Cad, cadaverine; Spd, spermidine; Spm, spermine) and agmatine (Agm) standard, respectively. *Trace amounts of putrescine were present as a contaminant in the agmatine standard reagent. (b) Comparison of MS/MS spectra of PEA standard and unidentified biogenic amine purified from culture supernatant of *T. nexilis*. (c and d) Comparison of chromatograms of PEA standard and unidentified biogenic amine purified from culture supernatant of *T. nexilis*: (c) Chromatograms obtained by reverse-phase HPLC (d) Chromatograms obtained by cation exchange HPLC. (e) PEA concentration in the culture supernatant of 32 species of the GAM culturable, dominant human gut bacteria.^47^ White and gray bars indicate PEA concentration in the culture supernatant in the growing and stationary phase, respectively. Data represent the mean ± SD of three individual experiments. See also Supplementary Figure S1.

Recently, we reported that 32 of the most predominant species of human indigenous gut microbiota were culturable in Gifu anaerobic medium (GAM)^47^ and evaluated polyamine biosynthesis and transport using this system.^48^ Reanalysis of the high-performance liquid chromatography (HPLC) chromatograms obtained in the polyamine study revealed that *Blautia hansenii, Clostridium asparagiforme, Tyzzerella nexilis, En. faecalis*, and *R. gnavus* produced unidentified biogenic amine in the culture supernatant (Figure 1a and Supplementary Figure S1). The retention time of the unidentified biogenic amine did not correspond to that of polyamines (putrescine, cadaverine, spermidine, spermine, and agmatine) (Figure 1a). To identify the unidentified biogenic amine, we purified this compound using an ion exchange chromatography from the culture supernatant of *T. nexilis*. The MS/MS spectra of the purified biogenic amine corresponded to that of PEA (Figure 1b). The retention time of the purified unidentified biogenic amine corresponded to PEA standard sample, in two different HPLC systems (Figure 1c and 1d). These results indicated that the unidentified biogenic amine is PEA.

Five species of the human dominant gut bacteria (*B. hansenii, C. asparagiforme, T. nexilis, En. faecalis*, and *R. gnavus*) produced PEA in their culture supernatants. The PEA concentrations in the culture supernatants in stationary phase were 240 μM for *B. hansenii*, 74 μM for *C. asparagiforme*, 447 μM for *T. nexilis*, 2,572 μM for *En. faecalis*, and 1,317 μM for *R. gnavus* (Figure 1e).

### Production of aromatic amines by PEA-producing gut bacteria in aromatic-amino-acids-defined medium

**Figure 2.**
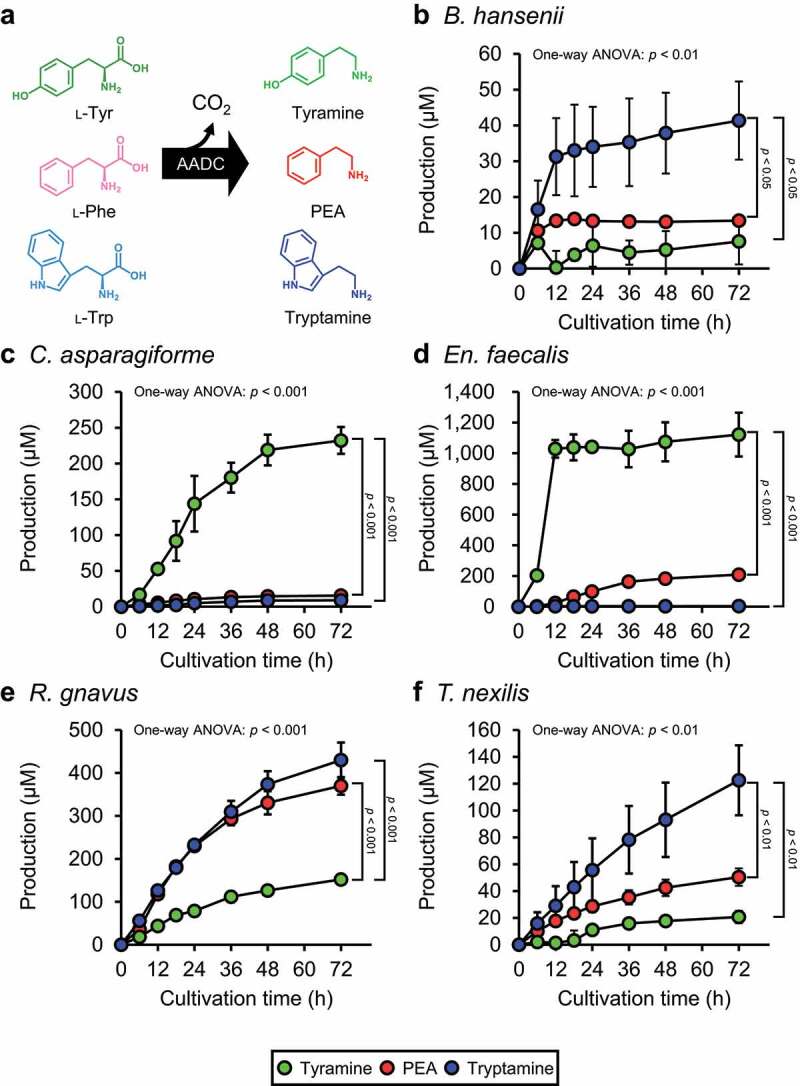
Aromatic amine production profile of identified PEA-producing gut bacteria. (a) Aromatic amino acid decarboxylase (AADC) decarboxylates proteinogenic aromatic amino acids (Tyr, Phe, and Trp) and generates corresponding aromatic amines (tyramine, PEA, and tryptamine). (b-f) Aromatic amine concentration in the culture supernatants of PEA-producing gut bacteria grown in AAAD medium: (b) *Blautia hansenii* (c) *Clostridium asparagiforme* (d) *Enterococcus faecalis* (e) *Ruminococcus gnavus* (f) *Tyzzerlla nexilis*. Green, red, blue circles indicate tyramine, PEA, and tryptamine concentrations in the culture supernatants, respectively. The “Production” value on the vertical axis was calculated by subtracting the amount of each aromatic amine originally contained in AAAD medium from the amount of aromatic amine at each time point. Data represent the mean ± SD of three or four individual experiments. The statistical significance of the PEA, tyramine, and tryptamine production at 72 h was determined using the one-way ANOVA post-hoc Tukey-Kramer test, and the *p*-values for the Tukey-Kramer test are shown. See also Supplementary Figure S2 and Supplementary Table S3.

It was reported that PEA was biosynthesized from Phe in the reaction catalyzed by AADC, a pyridoxal-5′-phosphate-dependent decarboxylase.^25,27^ AADC decarboxylates not only Phe, but also other proteinogenic aromatic amino acids (Tyr and Trp)^25,27^ (Figure 2a). The PEA-producing gut bacteria (*B. hansenii, C. asparagiforme, T. nexilis, En. faecalis*, and *R. gnavus*) possess the potential for the production of tyramine and tryptamine (Figure 2a). As the concentrations of the substrates of AADC: Phe, Tyr, and Trp, in GAM were different from each other (Supplementary Table S1), the production ability of each aromatic amine could not be quantitatively compared based on the concentration of PEA, tyramine, and tryptamine in the culture supernatant. Therefore, an aromatic-amino-acid-defined (AAAD) medium, where the concentrations of aromatic amino acids were adjusted to 1 mM, was prepared (Supplementary Table S2). The concentrations of the corresponding aromatic amines, PEA, tyramine, and tryptamine, in the culture supernatant of PEA-producing gut bacteria were analyzed.

All tested PEA-producing gut bacteria could grow in the AAAD medium (Supplementary Figure S2) and PEA was detected in the culture supernatants of four species (*B. hansenii, En. faecalis, R. gnavus*, and *T. nexilis*) (Figure 2b to 2f). Aromatic amine concentrations at all measured cultivation times were analyzed by repeated measures one-way ANOVA (Supplementary Table S3). For those aromatic amines with significantly different concentrations, values at 0 h and 72 h after inoculation were subjected to a post-hoc Tukey-Kramer test to verify whether they were significantly increased by the cultivation of the bacteria. Statistically significant aromatic amine production was observed, except for tyramine production by *B. hasenii* and tryptamine production by *En. faecalis* and *C. asparagiforme* (Figure 2b to 2d and Supplementary Table S3). Production of PEA in the culture supernatants of PEA-producing gut bacteria grown in AAAD medium was reached the maximum at 72 h after inoculation; the concentrations were 13 μM for *B. hansenii*, 15 μM for *C. asparagiforme*, 208 μM for *En. faecalis*, 370 μM for *R. gnavus*, and 50 μM for *T. nexilis*, (Figure 2b to 2f). Then, one-way ANOVA and post-hoc Tukey-Kramer test (Figure 2b to 2f) were performed to compare the concentrations of the three aromatic amines in each species after 72 h of cultivation. In *B. hansenii*, the concentration of tryptamine was significantly higher than that of tyramine and PEA, with no significant difference between the concentrations of PEA and tyramine (Figure 2b). In *C. asparagiforme*, the concentration of tyramine was significantly higher than that of PEA and tryptamine, with no significant difference between the concentrations of PEA and tryptamine (Figure 2c). In *En. faecalis*, the concentration of tyramine was significantly higher than that of PEA and tryptamine, with no significant difference between the concentrations of PEA and tryptamine (Figure 2d). Although there was no significant difference between the production of PEA and tryptamine in *En. faecalis*, the *p*-value obtained in the Tukey-Kramer test was 0.109, indicating a tendency toward higher PEA production than tryptamine (Figure 2d). In *R. gnavus*, the concentration of tryptamine and PEA were significantly higher than that of tyramine, with no significant difference between the concentrations of PEA and tryptamine (Figure 2e). In *T. nexilis*, the concentration of tryptamine was significantly higher than that of PEA and tyramine, with no significant difference between the concentrations of PEA and tyramine (Figure 2f).

### *Heterologous expression of* aadc *and* aadc *homologs*

Recombinant AADC proteins of *R. gnavus* (AADC_Rg_) and *En. faecalis* (AADC_Ef_) synthesize aromatic amines from aromatic amino acids, *in vitro*.^25,26^ The AADC of other three PEA-producing gut bacteria (*B. hansenii, C. asparagiforme*, and *T. nexilis*) were not identified experimentally; however, BLASTP analysis^49^ using AADC_Rg_ as the query protein showed that AADC homologs were present in *B. hansenii* (BLAHAN_06497, hereafter referred to as *aadc*_Bh_), *C. asparagiforme* (CLOSTASPAR_05940, hereafter referred to as *aadc*_Ca_), and *T. nexilis* (CLONEX_01451, hereafter referred to as *aadc*_Tn_). To determine the productivity of aromatic amine by AADC and AADC homologs of PEA-producing gut bacteria, the genes or its homologs encoding AADCs: *aadc*_Rg_, *aadc*_Ef_, *aadc*_Bh_, *aadc*_Ca_, and *aadc*_Tn_ were cloned into overexpression vectors and introduced into *E. coli* and the concentration of aromatic amines in the culture supernatants of *E. coli* strains were measured.

**Figure 3.**
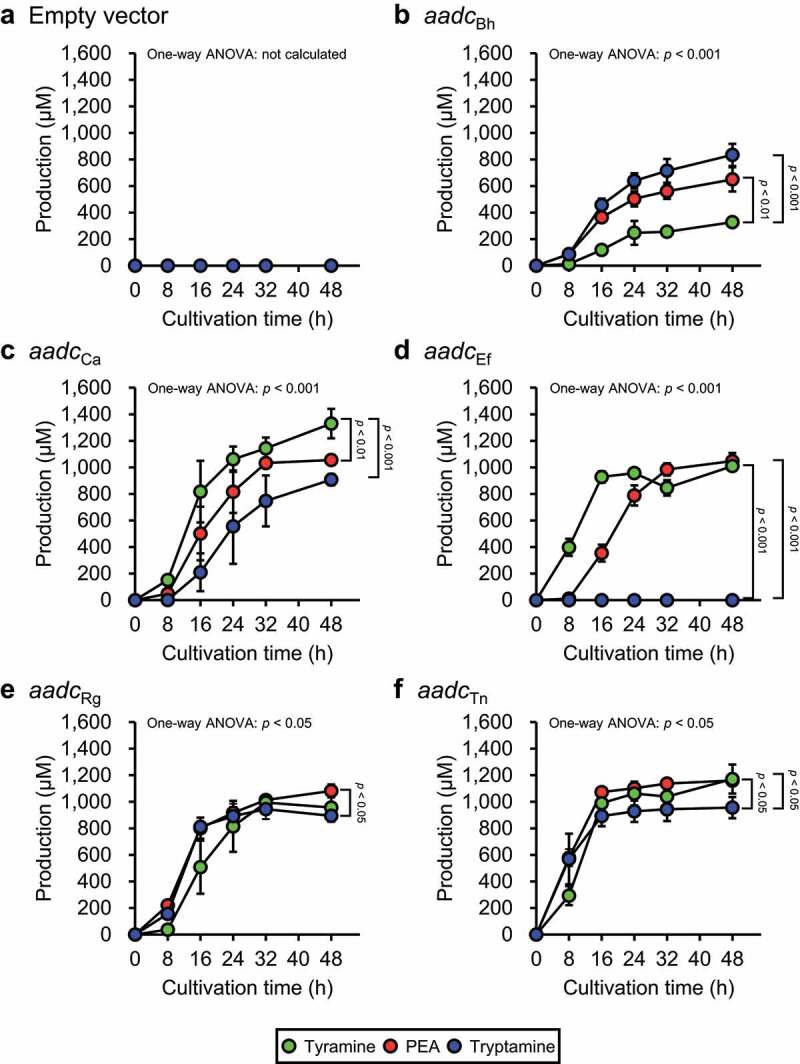
Aromatic amine concentration in the culture supernatants of *E. coli* harboring *aadc* or *aadc* candidate gene of PEA-producing gut bacteria. *E. coli* transformants were grown in M9AAA-medium and the aromatic amine concentrations in the culture supernatants were measured: (a) YS297 (empty vector), (b) YS389 (expressing *aadc*_Bh_), (c) YS300 (expressing *aadc*_Ca_), (d) YS317 (expressing *aadc*_Ef_), (e) YS298 (expressing *aadc*_Rg_), and (f) YS299 (expressing *aadc*_Tn_). Green, red, and blue circles indicate tyramine, PEA, and tryptamine concentrations in the culture supernatants, respectively. Data represent the mean ± SD of three individual experiments. The statistical significance of the PEA, tyramine, and tryptamine production at 48 h was determined using the one-way ANOVA post-hoc Tukey-Kramer test, and the *p*-values for the Tukey-Kramer test are shown. See also Supplementary Table S4.

*E. coli* harboring the empty vector (YS297) did not produce any of the aromatic amines (Figure 3a), whereas the *E. coli* strains heterologously expressing the *aadc* and *aadc* homologs produced more than 600 μM PEA, 800 μM tryptamine, and 300 μM tyramine at 48 h after inoculation (Figures 3b to 3f), confirming that the production of aromatic amines was due to the heterologous expression of the *aadc* and *aadc* homologs. Aromatic amine concentrations at all measured cultivation times were analyzed by repeated measures one-way ANOVA (Supplementary Table S4). For the aromatic amines with significantly different concentrations, values at 0 h and 48 h after inoculation were subjected to a post-hoc Tukey-Kramer test to verify whether they were significantly increased by the cultivation of the bacteria. Statistically significant aromatic amine production was observed, except for tryptamine production by YS317 harboring *aadc*_Ef_ (Figure 3d and Supplementary Table S4). One-way ANOVA and post-hoc Tukey-Kramer test (Figure 3b to 3f) were performed to compare the concentrations of the three aromatic amines in the culture supernatant of each *E. coli* strain after 48 h of cultivation. In YS389 harboring *aadc*_Bh_, the concentration of tryptamine and PEA was significantly higher than that of tyramine (Figure 3b). In contrast to the results obtained from *B. hansenii* cultured in AAAD medium (Figure 2b), the results obtained from YS389 showed a significantly higher concentration of PEA than tyramine at the end of cultivation (Figure 3b). In YS300 harboring *aadc*_Ca_, the concentration of tyramine was significantly higher than that of PEA and tryptamine (Figure 3c). Similar results were obtained when *C. asparagiforme* was cultured in AAAD medium (Figure 2c). YS389, YS300, YS298, and YS299 harboring *aadc*_Bh_ (Figure 3b), *aadc*_Ca_ (Figure 3c), *aadc*_Rg_ (Figure 3e), and *aadc*_Tn_ (Figure 3f), respectively, produced tryptamine, whereas YS317 harboring *aadc*_Ef_ did not produce tryptamine during the cultivation period. In contrast to the culture supernatant of *En. faecalis* in AAAD medium (Figure 2d), the culture supernatant of YS317 harboring *aadc*_Ef_ showed no significant difference in the final concentrations of PEA and tyramine, although the concentration of PEA reached its maximum after tyramine (Figure 3d). In YS298 harboring *aadc*_Rg_, the concentration of PEA was significantly higher than that of tryptamine (Figure 3e). The concentrations of tryptamine and tyramine were significantly different in *R. gnavus* cultivated in AAAD medium (Figure 2e) but not in YS298 (Figure 3e). In contrast to *T. nexilis* cultivated in AAAD medium (Figure 2f), the culture supernatant of YS299 harboring *aadc*_Tn_ contained a significantly higher concentration of PEA and tyramine than tryptamine (Figure 3f).

### *Correlation of gut bacterial* aadc *copy number and PEA production ability in human feces*

Heterologous expression experiments indicated that *aadc* candidates: *aadc*_Bh_, *aadc*_Ca_, *aadc*_Tn_, were involved in PEA production (Figure 3b to 3f), in addition to *aadc*_Ef_ and *aadc*_Rg,_ whose role in PEA production was already reported. However, it was still unclear whether *aadc* of PEA-producing gut bacteria produce PEA in the human colonic lumen. Therefore, the correlation between the copy number of *aadc*_Ef_, *aadc*_Rg_, *aadc*_Bh_, and *aadc*_Tn_ and the amount of PEA produced when incubated with 1 mM Phe was analyzed, using human fecal samples. *C. asparagiforme* produced 254 μM of tyramine in AAAD medium; however, PEA production was extremely low (15 μM, Figure 2c), and therefore, we excluded *aadc*_Ca_ from the analysis.

**Figure 4.**
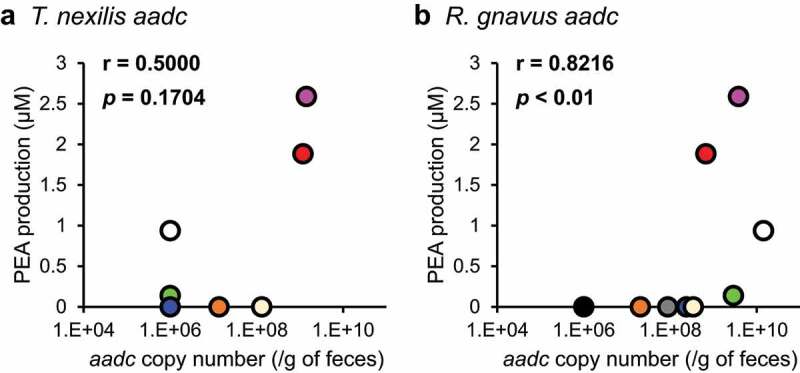
PEA production in feces is associated with *aadc* of *R. gnavus*. Nine human feces were separately incubated with or without 1 mM Phe. PEA production was calculated from the difference between PEA concentration when incubated with 1 mM Phe and that without 1 mM Phe. Copy number of *aadc* of *T. nexilis* and *R. gnavus* were determined using qPCR. (a) Correlation between PEA production and copy number of *aadc* of *T. nexilis*. (b) Correlation between PEA production and copy number of *aadc* of *R. gnavus*. Spearman’s rank correlation test was used for the correlation analysis (r = correlation coefficient). The copy number of the *aadc* gene in samples where the *aadc* gene was below the detection limit in our system was set as 10^6^ copies/g of feces. Donors are distinguished by color; the same color in (a) and (b) indicates the same donor.

The concentrations of PEA produced in the fecal culture ranged from 0 to 2.6 μM (Figure 4a and 4b). *aadc*_Tn_ was detected in 44% of donors (4/9); *aadc*_Rg,_ in 88% (8/9), while *aadc*_Ef_ and *aadc*_Bh_ were not detected by the qPCR assay. A significant correlation was observed between PEA production and *aadc*_Rg_ (r = 0.8216 and *p*= 0.0066, Figure 4b), but not between PEA production and *aadc*_Tn_ (r = 0.5000 and *p*= 0.1704, Figure 4a).

### *Influence of gut bacterial* aadc *on colonic aromatic amine production in colon and serotonin level*

**Figure 5.**
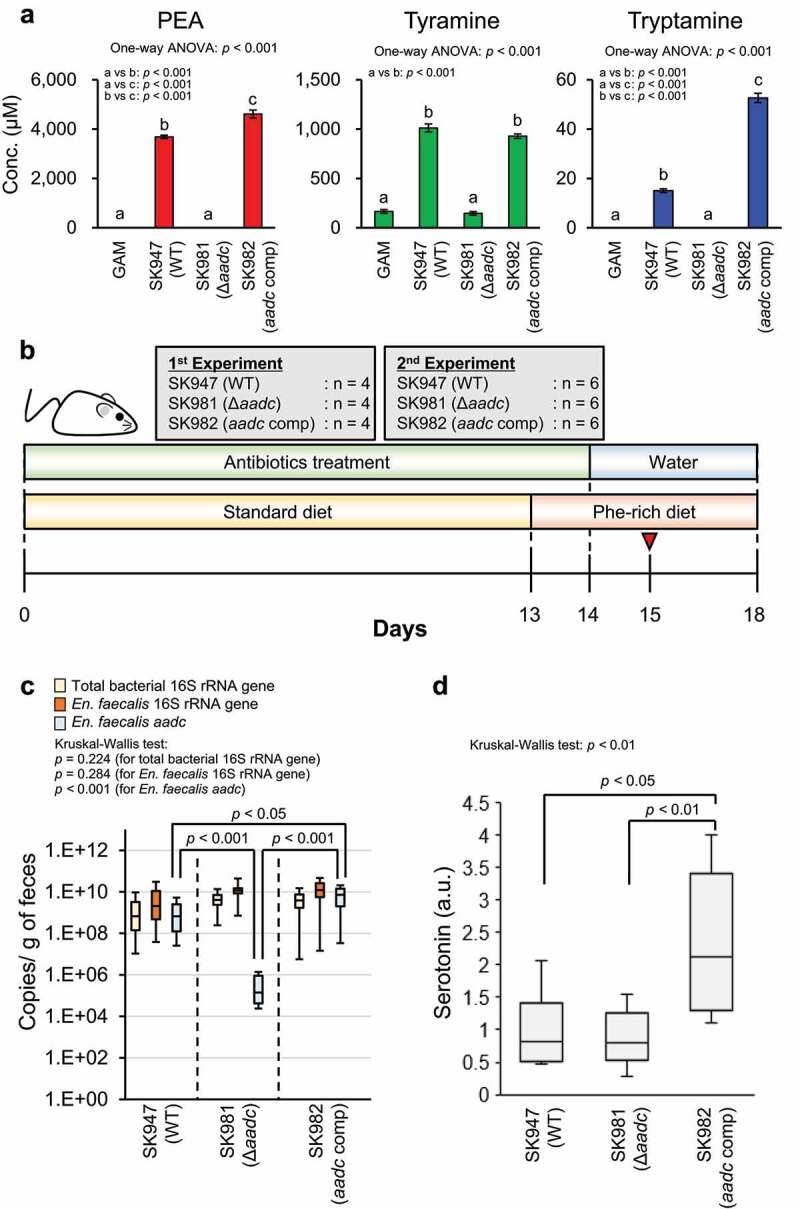
*aadc* modulates colonic serotonin levels. (a) Effects of deletion and complementation of *aadc* on the aromatic amine production of *En. faecalis in vitro. En. faecalis* WT (SK947), Δ*aadc* (SK981), and *aadc* complemented (SK982) strains were pre-cultured at 37°C in GAM, containing chloramphenicol (10 μg/mL) for 18 h in an anaerobic chamber. Each bacterial preculture was inoculated at a final optical density (OD_600_) of 0.03 in GAM. Strains were cultured at 37°C in GAM with chloramphenicol (10 μg/mL) for 72 h in an anaerobic chamber. Aromatic amine concentration in the culture supernatants was quantified using HPLC. Data represent the mean ± SD of three individual experiments. One-way ANOVA was performed to assess for significant differences in the aromatic amine concentrations between the groups, and the *p*-values for one-way ANOVA are indicated. Statistical significance between the strains was further analyzed by Tukey-Kramer test. The groups indicated by different letters were statistically different to each other, and the *p*-values between the groups indicated by the different letters are shown in each panel. (b) The feeding schedule for mice. Six-weeks-old female BALB/cCrSlc mice were given antibiotics in drinking water for two weeks to remove the indigenous bacteria. Mice were fed a standard diet for 13 d, then a Phe-rich diet for 5-days. *En. faecalis* (WT, Δ*aadc, aadc* complementation) (1 × 10^8^ cfu) was inoculated into mice on day 15, indicated by the red arrow (1 day after stopping antibiotics treatment). (c) *En. faecalis* colonization of mouse colon confirmed using qPCR. Statistical analysis was performed using Steel-Dwass test. No significant differences were observed between the groups, for total bacterial 16S rRNA gene and *En. faecalis* 16S rRNA gene. Two independent experiments were performed (n = 4 and 6 in each group, respectively), and data is shown in box plots with the median ± interquartile range. Statistical significance for each gene copy number between the groups was assessed by the Kruskal-Wallis test post-hoc Steel-Dwass test. The *p*-values for the Kruskal-Wallis test and Steel-Dwass test are shown. (d) Colonic serotonin levels in mice colonized with *En. faecalis*. Serotonin concentrations were measured using ELISA. The amount of serotonin in each mouse was normalized by the mean value obtained for WT colonized mice. Two independent experiments were performed (n = 4 and 6 in each group, respectively). The amounts are expressed as serotonin (a.u.). The data are shown by box plots, in which the horizontal line inside the box is the median. Statistical significance was assessed by the Kruskal-Wallis test post-hoc Steel-Dwass test. The *p*-values for the Kruskal-Wallis test and Steel-Dwass test are shown. See also Supplementary Figure S4.

We hypothesized that colonic luminal PEA, as with tyramine,^24^ induces serotonin production from EC cells via TAAR1. To confirm this hypothesis in experiments with BLAB/cCrSlc mice, we purchased and used the colons of BLAB/cCrSlc mice to evaluate *Taar1* expression in colon. RNA was extracted from the purchased colons of BALB/cCrSlc mice, PCR was performed using the reverse transcription product as a template with *Taar1*-specific primers,^50^ and the PCR product was sequenced. As a result, the expression of *Taar1* in the BALB/cCrSlc mouse colon was confirmed (Supplementary Figure S3). Next, to analyze the effects of indigenous bacterial PEA production in the intestinal lumen on the colonic serotonin production in the host at gut bacterial gene level, we selected *En. faecalis*, which can be genetically engineered, among the PEA-producing gut bacteria and generated *aadc*-deletion (SK981) and complementation (SK982) strains of *En. faecalis*. The production of aromatic amines was completely lost by the deletion of *aadc*, as previously described^29^ and the production of aromatic amines was recovered by the complementation of *aadc* (Figure 5a). The colonic serotonin levels of mice colonized with wild-type *En. faecalis* (SK947), Δ*aadc* (SK981), or *aadc*-complemented strain (SK982) were measured.

A schematic overview of the mice experiment is shown in Figure 5b. The number of *En. faecalis* was almost the same in the feces of mice colonized with wild type, ∆*aadc*, and *aadc*-complemented *En. faecalis*, suggesting that the colonization efficiency was not influenced by *aadc* deletion or complementation (Figure 5c). The fecal *aadc* copy number was significantly higher in mice colonized with *aadc*-complemented strain than in those colonized with wild-type *En. faecalis*. The fecal *aadc* copy number of mice colonized with ∆*aadc En. faecalis* was in the order of 1/3,000, compared to that of mice colonized with wild type *En. faecalis* (Figure 5c). Serotonin levels were significantly higher in the colon tissue of mice colonized with *aadc*-complemented strain than those colonized with wild-type *En. faecalis* or ∆*aadc En. faecalis*. However, no significant difference in the serotonin levels in the colon tissue was observed between mice colonized with wild-type *En. faecalis* and mice colonized with ∆*aadc En. faecalis* (Figure 5d). These data suggest that a high copy number of *aadc* (~10^10^ copy number/g of feces), which was also observed in some samples during our human fecal assays (Figure 4), induces colonic serotonin production (Figure 5c and 5d). However, PEA was not detected in the feces and the cecal contents of any of the mice. A small amount of tyramine was detected in the feces of mice colonized with wild-type and *aadc*-complemented *En. faecalis*, while no tyramine was detected in the feces of mice colonized with ∆*aadc En. faecalis* (Supplementary Figure S4A). These results indicate that gut bacterial *aadc* contributes to aromatic amine production in the host colonic lumen. However, no significant correlation between the fecal tyramine amounts and colonic serotonin amounts was observed in the groups (Supplementary Figure S4B).

### *Human AADC inhibitor decreases PEA production in* En. faecalis

**Figure 6.**
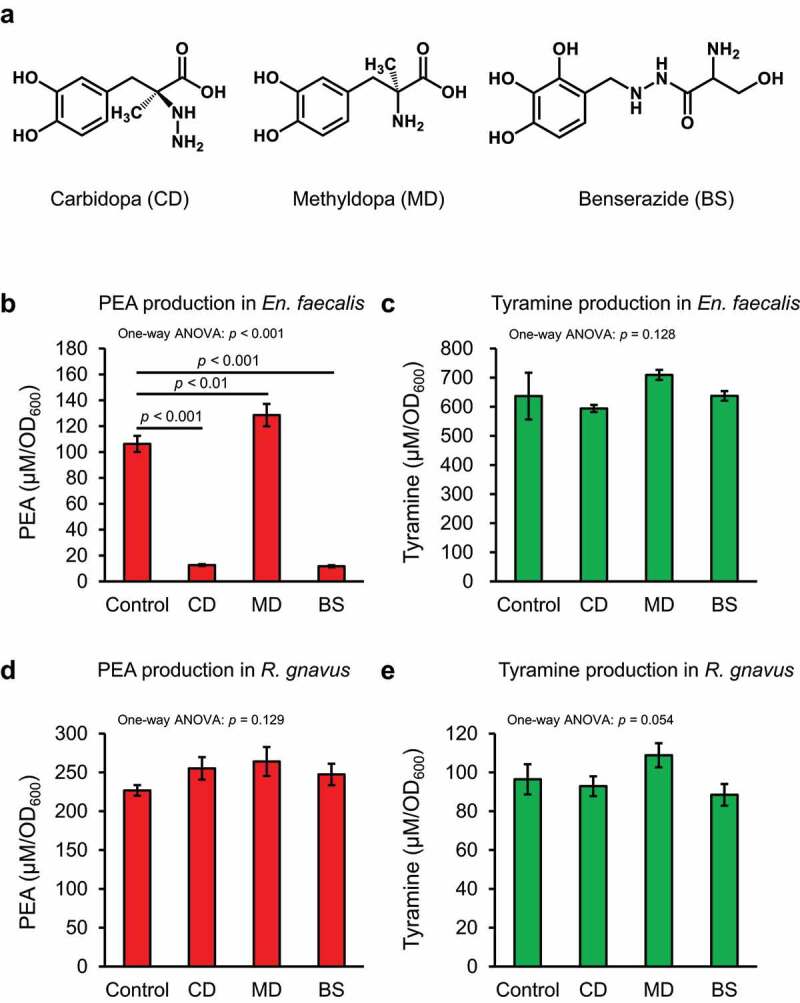
Human AADC inhibitors inhibit PEA production in *En. faecalis. En. faecalis* and *R. gnavus* were cultured with 1.5 mM human AADC inhibitor in AAAD medium for 24 h, and the concentration of PEA and tyramine in the culture supernatants were quantified using HPLC. PEA and tyramine concentrations were normalized to the OD_600_ values and shown as μM/OD_600_. (a) Structure of the tested human AADC inhibitors. (b) and (c) Effect of the human AADC inhibitors on PEA and tyramine production in *En. faecalis*, respectively. (d) and (e) Effect of the human AADC inhibitors on PEA and tyramine production in *R. gnavus*, respectively. Data represent the mean ± SD of three individual experiments. Statistical significance was assessed by one-way ANOVA post-hoc Dunnett’s test. The *p*-values for one-way ANOVA and Dunnett’s test are shown. See also Supplementary Figure S5.

Several human AADC inhibitors have been clinically used for the treatment of Parkinson’s disease,^51^ and these inhibitors may be candidates for drug repositioning. We evaluated whether human AADC inhibitors (carbidopa, methyldopa, and benserazide [Figure 6a]) could inhibit PEA production of *En. faecalis* and *R. gnavus*, which produced PEA *in vitro* (Figure 2d and 2e). Severe growth deficiency was not observed with any tested AADC inhibitors (Supplementary Figure S5A and S5B). PEA production in *En. faecalis* was strongly inhibited by carbidopa and benserazide, however methyldopa did not inhibit PEA production (Figure 6b and Supplementary Figure S5C). Surprisingly, tyramine concentration in the culture supernatant of *En. faecalis* did not change when treated with carbidopa and benserazide (Figure 6c and Supplementary Figure S5D). None of the tested human AADC inhibitors inhibited PEA production by *R. gnavus* (Figure 6d and Supplementary Figure S5E). Although carbidopa and benserazide significantly reduced tyramine production by *R. gnavus* (Supplementary Figure S5F), they also significantly reduced *R. gnavus* growth (Supplementary Figure S5B). Therefore, the tested inhibitors exerted no significant effects after the tyramine concentration of *R. gnavus* was normalized to bacterial growth (OD_600_) (Figure 6e).

## Discussion

Gut microbiota produce various metabolites in the colonic lumen, affecting the host physiology.^52^ Identification of gut bacterial species and genes responsible for metabolite production is essential for the optimization of the state of the intestinal environment. Some molecular mechanisms of metabolite production in the colonic lumen have been established at the genetic level,^53–55^ however, even for well-known metabolites, there are unidentified mechanisms modulating the production. Few studies have investigated between intestinal bacterial genes and the host physiology. In this study, we identified five PEA-producing bacteria from the GAM-culturable human dominant gut bacteria (Figure 1e) and revealed that *aadc* is indispensable for the production of PEA (Figures 3b-3f, and 5a). Our results suggested that gut bacterial AADC and its reaction product, PEA, participate in the peripheral serotonin production in the host. Furthermore, fecal culture and mouse experiments indicated that gut bacterial *aadc* contributes to aromatic amine production in the colon (Figure 4b and Supplementary Figure S4A).

AADC of *R. gnavus* was recently identified as a Trp decarboxylase, and studies using the recombinant AADC indicated that the catalytic efficiency (*k*_cat_/*K*_m_) for Trp is 1,000-fold higher than that for Phe.^25^ However, in our experiment using the AAAD medium containing 1 mM of Trp, Tyr, and Phe, *R. gnavus* produced statistically significantly more tryptamine than PEA, but the difference was 1.2-fold, which was much smaller than the difference expected from *k*_cat_/*K*_m_ value (Figure 2e). *B. hansenii* (Figure 2b) *and T. nexilis* (Figure 2f), which were considered tryptamine-producing gut bacteria^25^ produced not only tryptamine, but also PEA and/or tyramine in the AAAD medium. Aromatic amine productivity of *E. coli* transformants expressing each *aadc* from PEA-producing gut bacteria in M9AAA-medium were partially but not entirely consistent with the PEA-producing gut bacteria in AAAD medium (Figures 2b to 2f and 3b to 3f). These differences were hypothesized to be due to the different abilities of *E. coli* and PEA-producing gut bacteria to uptake aromatic amino acids or to release aromatic amines. These results suggested that the production of metabolites secreted by bacterial cells is influenced not only by the activity of the enzymes, but also by that of transporters. A significant correlation was observed between *aadc*_Rg_ and the production of PEA in fecal culture (Figure 4b). Therefore, our results suggest that PEA production was not a side reaction, but rather the main reaction catalyzed by AADC in the intestinal lumen. On the other hand, no significant correlation was observed between *aadc*_Tn_ and the PEA production (Figure 4b). This could possibly be because of the lower number of the fecal samples possessing *aadc*_Tn_. In addition, *in vitro* analysis showed that *R. gnavus* produced more PEA than *T. nexilis* (Figure 2e and 2f), suggesting that the contribution of *aadc*_Rg_ is significant compared to that of *aadc*_Tn,_ in the PEA production in colonic lumen. We also observed that while possessing the same level of *aadc*_Rg_ in the feces, the fecal samples differ in PEA production (Figure 4b). Gut bacteria reductively metabolize aromatic amino acids to aromatic lactate, aromatic acrylate, and aromatic propionate by aromatic lactate dehydrogenase, phenyl lactate dehydrogenase, phenyl lactate dehydratase, or acyl-CoA dehydrogenase after deamination.^10,55^ Therefore, we speculate that the amount of the genes related to reductive aromatic amino acid metabolism pathway affects PEA production.

Peripheral serotonin is produced by EC cells^56^ and its production heavily relies on gut bacteria.^24^ Physiologically active metabolites derived from gut bacteria, such as short-chain fatty acids (acetic acid and butyric acid),^57^ and deoxycholate^24^ stimulate peripheral serotonin production. Intrarectally injected tyramine induces peripheral serotonin secretion in mouse.^24^ EC cells express a variety of receptors, including TAAR1;^58^ like tyramine, PEA is also a ligand of TAAR1.^35^ Colonic *Taar1* expression has been reported in humans,^37^ and BALB/cCrSlc mice of the same strain as used in the experiment to assess the effect of gut bacterial PEA on the colonic serotonin, was also expressing *Taar1* in the colon (Supplementary Figure S3). Furthermore, activation of TAAR1 induced serotonin production in a cell model.^35^ In this study, colonic serotonin levels of mice colonized with *aadc*-complemented *En. faecalis* were higher than those in mice colonized with wild-type *En. faecalis* and Δ*aadc En. faecalis* (Figure 4d). There are two possible sources of phenylalanine in the mouse colon: diet and metabolic activity of gut bacteria. Phe, a substrate for AADC, has been detected in mice colon and its amount increase in response to feeding a high-fat diet.^59^ Therefore, it is possible that PEA is generated in the murine colonic lumen by the reaction catalyzed by AADC. However, in our experiments, PEA was detected neither in the feces nor in the cecal contents of mice. A possible reason for this is that PEA produced by gut bacteria in the intestinal tract after inducing serotonin production in mice was rapidly degraded by a reaction catalyzed by monoamine oxidase-B (MAO-B)^60^ and was undetectable by the time of analysis. Actually, the expression of *Mao-b* in the colon of BALB/cCrSlc mice of the same strain as used in the experiment to assess the effect of gut bacterial PEA on the colonic serotonin was confirmed by PCR (Supplementary Figure S3). The reason why tyramine, but not PEA, was detected (Supplementary Figure S4A) in the colonic lumen could be that the *K*_m_ of MAO-B, which reportedly is expressed in the intestinal tract,^24^ to tyramine is described to be 290-fold higher than that to PEA.^61^ Therefore, it is likely that only PEA was preferentially degraded by MAO-B. In the present study, a maximum of 41.8 pmol of tyramine per mg of feces was detected (Supplementary Figure S4A), but tyramine concentration in the feces did not correlate significantly with serotonin production in the intestinal tract (Supplementary Figure S4B). On the contrary, Yano *et al*. demonstrated that injection of about 20 μmol of tyramine into the mouse colonic lumen activated colonic serotonin production.^61^ Given a mouse fecal mass of 100 to 200 mg,^62,63^ the amount of tyramine detected in the colon in the present study was 4.2 to 8.4 nmol, much less than the dose given by Yano *et al*.^24^ This could explain why tyramine amounts in the intestinal lumen did not correlate with serotonin production in the intestinal tract; thus, this study does not exclude the theory that tyramine promotes serotonin production^24^ in the colon.

Peripheral serotonin is an important regulator and is implicated in several diseases, such as osteoporosis,^64^ diarrhea-predominant irritable bowel syndrome,^65^ celiac disease,^66^ inflammatory bowel disease,^67^ and obesity.^68^ Marketed drugs: Carbidopa and benserazide, developed as human AADC inhibitor, significantly inhibited PEA production in *En. faecalis* (Figure 6b). However, none of these inhibitors inhibited tyramine production by *En. faecalis* and PEA and tyramine production by *R. gnavus* (Figure 6c, 6d, and 6e). AADC_Ef_ is capable of decarboxylating L-DOPA, while AADC_Rg_ cannot.^29^ The human AADC inhibitor is an analogue of L-DOPA, and therefore likely fits into the substrate pocket of AADC_Ef_ to inhibit PEA production. However, the low affinity of AADC_Rg_ for L-DOPA might have abrogated the inhibitory effect. (*S*) -α-fluoromethyl tryptophan inhibits AADC of *R. gnavus*,^25^ while (*S*)-α-fluoromethyl tyrosine inhibits AADC of *En. faecalis* . Therefore, (*S*)-α-fluoromethyl derivatives, such as (*S*)-α-fluoromethyl phenylalanine may be promising as inhibitors of PEA synthesis by AADC of gut bacteria.

Here, we showed that gut bacterial AADC contributes colonic serotonin production, and that two commercial AADC inhibitors can inhibit PEA production by *En. faecalis*. However, our study had some limitations. First, we used BALB/cCrSlc mice as a model. This strain possesses a mutation in the *tryptophan hydroxylase-2* gene, which is responsible for central serotonin biosynthesis.^69,70^ BALB/cCrSlc mice has not been reported to exhibit a mutation in *tryptophan hydroxylase-1*, which is responsible for the peripheral serotonin biosynthesis evaluated in the present study. The determined colonic serotonin level (0.49 to 6.8 μg/g of colonic tissue) was comparable to that in previous studies using C57BL/6 mice.^71^ However, other models are needed to assess PEA effects on serotonin, including in the central nervous system. Second, we evaluated the effects of human AADC inhibitors on gut bacterial aromatic amine production with 1.5 mM. This dosage has technical and toxicological limitations, e.g., to reach 1.5 mM of carbidopa in the intestinal tract, a minimum of 1 g must be ingested.^72^ To solve this problem, it is necessary to develop efficient drug delivery methods to the intestinal tract; for example, the administration of carbidopa in enteric capsules and the development of effective gut bacterial AADC inhibitors that are not absorbed in the intestine should be considered. In the future, selective and efficient inhibition of gut bacterial AADC could lead to the prevention and treatment of diseases involving peripheral serotonin.

## Conclusion

The present study presented five PEA-producing gut bacteria species and determined that aromatic amine production from proteinogenic aromatic amino acids depends on AADC activity. Human fecal culture experiments revealed a significant positive correlation between *aadc* of *R. gnavus* and PEA production, suggesting that *R. gnavus* contributes to PEA production in the human colonic lumen. Furthermore, gut bacterial *aadc* activity upregulated colonic serotonin levels in a mice model treated with genetically modified *En. faecalis*. Finally, we revealed that the human AADC inhibitors carbidopa and benserazide prevented PEA production by *En. faecalis*. This study shows that AADC in gut bacteria may be a potential target in the prevention and treatment of diseases involving peripheral serotonin.

## Materials and Methods

### Chemicals

Phenylethylamine hydrochloride (Cat# P0086), tryptamine (Cat# T0890), tyramine hydrochloride (Cat# A0303), L-tyrosine (Cat# T0550), carbidopa monohydrate (Cat# C2450), methyldopa sesquihydrate (Cat# D1817), and benserazide hydrochloride (Cat# B4108) were purchased from Tokyo Chemical Industry (Tokyo, Japan). L-Phenylalanine (Cat# 169–01303), Amino Acids Mixture Standard Solution, Type H (Cat# 013–08391) were obtained from FUJIFILM Wako Pure Chemical (Osaka, Japan). L-Tryptophan (Cat# 35607–74) was obtained from Nacalai Tesque (Kyoto, Japan). Other reagents of analytical grade were from FUJIFILM Wako Pure Chemical, Nacalai Tesque (Kyoto, Japan), and Sigma-Aldrich (St. Louis, MO, USA).

### Bacterial strains

The strains used in this study are listed in Table 1. Bacteria were obtained from the Japan Collection of Microorganisms (JCM), the American Type Culture Collection (ATCC), and the German Collection of Microorganisms and Cultures (DSMZ).

**Table 1.** Bacterial strains, plasmids, and primers used in this study.

Strain, plasmid, or primer	Description, genotype, or sequence of primers (5’ to 3’)	Purpose	Source or reference
*Escherichia coli*			
DH5α		Used as the host for plasmid construction	Laboratory stock
BL21(DE3)		Used as the host for heterologous expression of *aadc*	Novagen
YS297	pCDF23/BL21 (DE3)		This study
YS298	pYS295/BL21 (DE3)		This study
YS299	pYS294/BL21 (DE3)		This study
YS300	pYS296/BL21 (DE3)		This study
YS317	pYS316/ BL21 (DE3)		This study
YS389	pYS388/BL21 (DE3)		This study
Gut bacteria			
*Blautia hansenii*	JCM 14655 ^T^		Japan Collection ofMicroorganism
*Clostridium asparagiforme*	DSM 15981 ^T^		German Collection of Microorganisms and Cultures
*Tyzzerella nexilis*	ATCC 27757 ^T^		American Type Culture Collection
*Enterococcus faecalis*	ATCC 700802		American Type Culture Collection
*Ruminococcus gnavus*	ATCC 29149 ^T^		American Type Culture Collection
SK947	pLZ12/*En. faecalis*		^53^
SK981	pLZ12/*En. faecalis* Δ*aadc*_Ef_		This study
SK982	pLZ12_*aadc*_Ef_^+^/*En. faecalis* Δ*aadc*_Ef_		This study
Plasmids			
pUC19	ColE1 replicon *bla*^+^		^73^
pYS369	pUC19 harboring 16S rRNA gene of *En. faecalis*	Used as the template to generate a standard curve of bacterial and *En. faecalis* 16S rRNA genes in qPCR.	This study
pYS409	pUC19 harboring *aadc*_Ef_ (*aadc* of *En. faecalis*, EF_0634)	Used as the template to generate a standard curve of *En. faecalis aadc* in qPCR.	This study
pCDF23	CDF replicon *aadA*^+^ *lacI*	Used as the expression vector for *aadc* in *E. coli*	^74^
pYS294	pCDF23 harboring *aadc*_Tn_ (candidate *aadc* of *T. nexilis*, CLONEX_01451)		This study
pYS295	pCDF23 harboring *aadc*_Rg_ (*aadc* of *R. gnavus*, RUMGNA_01526)		This study
pYS296	pCDF23 harboring *aadc*_Ca_ (candidate *aadc* of *C. asparagiforme*, CLOSTASPAR_05940)		This study
pYS316	pCDF23 harboring *aadc*_Ef_ (*aadc* of *En. faecalis*, EF_0634)		This study
pYS388	pCDF23 harboring *aadc*_Bh_ (candidate *aadc* of *B. hansenii*, BLAHAN_06497)		This study
pLZ12	pSH71 replicon *cat*^+^	Used for gene complementation of *En. faecalis*	^75^
pLZ12_*aadc*^+^	pSH71 replicon *cat*^+^*aadc*_Ef_	Used for *aadc*_Ef_ complementation for *En. faecalis*	This study
pLT06	*repA*-ts replicon *pheS*^+^-*cat*^+^-*lacZ*^+^	Used for gene deletion of *En. faecalis*	^76^
pLT06_*aadc*^+^	*repA*-ts replicon *pheS*^+^-*cat*^+^-*lacZ*^+^ Δ*aadc*_Ef_	Used for *aadc*_Ef_ deletion of *En. faecalis*	This study
Primers			
Ef_tdc_Fw(NdeI)	AAGGAGATATACATATGAATGCAAAATCTAATTCA	Used for amplification of *aadc*_Ef_ to clone into NdeI and XhoI sites of pCDF23	This study
Ef_tdc_Rv(XhoI)	GGTGGTGGTGCTCGAGTTATTTTACGTCGTAAATTTG	Used for amplification of *aadc*_Ef_ to clone into NdeI and XhoI sites of pCDF23	This study
RUM_PEA_Fw(NdeI)	AAGGAGATATACATATGATCGGAACCGAATATATTTT	Used for amplification of *aadc*_Rg_ to clone into NdeI and XhoI sites of pCDF23	This study
RUM_PEA_Rv(XhoI)	GGTGGTGGTGCTCGAGTTAAGCTTTTTTCATTTCC	Used for amplification of *aadc*_Rg_ to clone into NdeI and XhoI sites of pCDF23	This study
CNEX_PEA_Fw(NdeI)	AAGGAGATATACATATGATTAACAGCGAAAATGTATTA	Used for amplification of *aadc*_Tn_ to clone into NdeI and XhoI sites of pCDF23	This study
CNEX_PEA_Rv(XhoI)	GGTGGTGGTGCTCGAGTTAGCTATTTTGTTTTTTCATATCTGC	Used for amplification of *aadc*_Tn_ to clone into NdeI and XhoI sites of pCDF23	This study
CASPA_PEA_Fw(NdeI)	AAGGAGATATACATATGGATTCTTTCATGGAAGCGCAG	Used for amplification of *aadc*_Ca_ to clone into NdeI and XhoI sites of pCDF23	This study
CASPA_PEA_Rv(XhoI)	GGTGGTGGTGCTCGAGTTAAGCCGTCCGGCACTGTC	Used for amplification of *aadc*_Ca_ to clone into NdeI and XhoI sites of pCDF23	This study
Bhan_PDC_Fw(NdeI)	AAGGAGATATACATATGATAAACAGTGAAAATATTTT	Used for amplification of *aadc*_Bh_ to clone into NdeI and XhoI sites of pCDF23	This study
Bhan_PDC_Rv(XhoI)	GGTGGTGGTGCTCGAGTTATTTCATATCTTTATAAAG	Used for amplification of *aadc*_Bh_ to clone into NdeI and XhoI sites of pCDF23	This study
7 F	AGAGTTTGATYMTGGCTCAG	Used for amplification of 16S rRNA gene of *En. faecalis* to clone into SmaI site of pUC19	^77^
1510 R	ACGGYTACCTTGTTACGACTT	Used for amplification of 16S rRNA gene of *En. faecalis* to clone into SmaI site of pUC19	^77^
pUC_EFtdc_SmaI_Fw	GTGAATGCAAAATCTAATTC	Used for amplification of *aadc*_Ef_ to clone into SmaI site of pUC19	This study
pUC_EFtdc_SmaI_Rv	TTATTTTACGTCGTAAATTTG	Used for amplification of *aadc*_Ef_ to clone into SmaI site of pUC19	This study
U16SRT-F	ACTCCTACGGGAGGCAGCAGT	Used for quantification of total bacterial 16S rRNA gene using qPCR	^78^
U16SRT-R	TATTACCGCGGCTGCTGGC	Used for quantification of total bacterial 16S rRNA gene using qPCR	^78^
Ef_16S_72 F	CCGAGTGCTTGCACTCAATTGG	Used for quantification of *En. faecalis* 16S rRNA gene using qPCR	^79^
Ef_16S_210 R	CTCTTATGCCATGCGGCATAAAC	Used for quantification of *En. faecalis* 16S rRNA gene using qPCR	^79^
Ef_tdc_qPCR_Fw	CTGCTGATATTATCGGTATCGGTT	Used for quantification of *aadc*_En_ using qPCR	^80^
Ef_tdc_qPCR_Rv	GTAGTTATGGTCAACTGGTACTGGG	Used for quantification of *aadc*_En_ using qPCR	^80^
Cnex_aadc_qPCR6_Fw	GCTCTCCGTGAATTAGATC	Used for quantification of *aadc*_Tn_ using qPCR	This study
Cnex_aadc_qPCR6_Rv	GCTTCTTCGCTTATTTCATCGG	Used for quantification of *aadc*_Tn_ using qPCR	This study
Rgna_aadc_qPCR2_Fw	AACCGGGCTTGCTGACAGTA	Used for quantification of *aadc*_Rg_ using qPCR	This study
Rgna_aadc_qPCR2_Rv	CGTACGTCTGGAAGAGCCATTT	Used for quantification of *aadc*_Rg_ using qPCR	This study
Bhan_aadc_qPCR1_Fw	CTCAGGCAGGATTTGGTGAAA	Used for quantification of *aadc*_Bh_ using qPCR	This study
Bhan_aadc_qPCR1_Rv	GCCATGGAACCTCCGCTTA	Used for quantification of *aadc*_Bh_ using qPCR	This study
for_Del_ddc_1_F	TGCAAATTGGTGGCGCTGAT	Used for amplification of 1,000 bp upstream from start codon of *aadc*_Ef_	This study
for_Del_ddc_2_5P_R	AACTTACACCCAAACGGCTA	Used for amplification of 1,000 bp upstream from start codon of *aadc*_Ef_	This study
for_Del_ddc_3_5P_F	TTGAATCTTAAACGGAAAAAGAAATG	Used for amplification of 1,000 bp downstream from stop codon of *aadc*_Ef_	This study
for_Del_ddc_4_R	TGGTTGTGTAATGTTAGACAATTC	Used for amplification of 1,000 bp downstream from stop codon of *aadc*_Ef_	This study
pLT06-EcoRI_D_ddc_F	TACCGAGCTCGAATTCTGCTTTAAGGTGGCGCTGAT	Used for fusion of 1,000 bp upstream of from start codon of *aadc*_Ef_ and 1,000 bp downstream from stop codon of *aadc*_Ef_ using overlap PCR and cloning into EcoRI site of pLT06	This study
pLT06-EcoRI_D_ddc_R	TACCGAGCTCGAATTCTGGTTGTGTAATGTTAGACAATTC	Used for fusion of 1,000 bp upstream of from start codon of *aadc*_Ef_ and 1,000 bp downstream from stop codon of *aadc*_Ef_ using overlap PCR and cloning into EcoRI site of pLT06	This study
C_ddc+0.5K_F_pLZ_Bam	GAATTCATTAGGATCCCAAGGGTTAGAACATGTGCC	Used for amplification of 500 bp upstream from start codon of *aadc*_Ef_ and *aadc*_Ef_ to clone into BamHI site of pLZ12	This study
C_ddc+0.5K_R_pLZ_Bam	AGGAAGATCTGAATCCTTACATTCGACTGCCTCTTACCC	Used for amplification of 500 bp upstream from start codon of *aadc*_Ef_ and *aadc*_Ef_ to clone into BamHI site of pLZ12	This study
Pr_GYS4	GCTTTCATTTCCATTGACCGCTAC	Used for *Taar1* expression analysis	^50^
Pr_GYS5	ATAGAAGGAAGTCATGAACGCCAG	Used for *Taar1* expression analysis	^50^
Pr_GYS6	CCATCACCATCTTCCAGGAGCGAG	Used for *Gapdh* amplification	^50^
Pr_GYS7	CACAGTCTTCTGGGTGGCAGTGAT	Used for *Gapdh* amplification	^50^
Pr_GYS8	GGAATCCTGTGGCATCCATGAAAC	Used for *Act-b* amplification	^81^
Pr_GYS9	TAAAACGCAGCTCAGTAACAGTCCG	Used for *Act-b* amplification	^81^
Pr_GYS12	GTTGAGCGGCTGATACACTTT	Used for *Mao-b* expression analysis	Primer Bank (ID 257196227c3)
Pr_GYS13	GAGCGTGGCAATCTGCTTTG	Used for *Mao-b* expression analysis	Primer Bank (ID 257196227c3)

Notes: The underlined nucleotides indicate the overlapping regions used for In-Fusion cloning.

### High-performance liquid chromatography (HPLC)

Quantification of aromatic amines and aromatic amino acids were carried out using HPLC. PEA was quantified by the post-column labeling method as describe previously.^82^ Briefly, PEA was separated using a cation-exchange column (#2619PH, Hitachi, Tokyo, Japan) in normal-phase mode, and derivatized with *o*-phthalaldehyde and detected using fluorescence detector (λ_ex_: 340 nm and λ_em_: 435 nm). Tyramine and tryptamine were separated in reverse-phase mode using a Discovery HS-F5 column (4.6 × 250 mm, 5 μm, SUPECLCO, Bellefonte, PA, USA) at 35°C. The elution was carried out at a flow rate of 0.4 mL/min with 10 mM ammonium formate (pH 3.0) and acetonitrile using the following gradient program: the concentration of acetonitrile was linearly increased from 3 to 27% during 0‒22 min, and linearly increased from 27 to 66% during 22–80 min, increased to 100% during 80‒81 min, maintained at 100% during 81‒86 min, returned to 3% during 86‒87 min, and maintained at 3% during 87‒102 min. In this analytical system, the retention time for Tyr, tyramine, Trp, and tryptamine were 18.5, 28, 29.3, and 51 min, respectively. The elution was monitored based on the fluorescence with excitation at 280 nm and emission at 325 nm, using a Waters 2475 Multi-wavelength Fluorescence detector (Waters, Milford, MA, USA). Phe concentration was quantified using HPLC equipped with a cation-exchange column (#2619, Hitachi). The temperature of the column oven was maintained at 70°C. Buffer-A (23 mM sodium citrate, 96.8 mM sodium chloride, 84.7 mM citric acid, 13% ethanol, and 0.0001% caprylic acid), buffer-B (26.3 mM sodium citrate, 121 mM sodium chloride, 105 mM citric acid, 0.02% ethanol, and 0.0001% caprylic acid), buffer-C (90 mM sodium citrate, 930 mM sodium chloride, 104 mM citric acid, and 0.0001% caprylic acid), and buffer-D (200 mM sodium hydroxide, 10% ethanol, and 0.0001% caprylic acid) were used as the mobile phase, at a flow rate of 0.4 mL/min. The gradient program was as follows: buffer-A was kept at 100% during 0–13 min. The buffer was changed to buffer-B and the proportion was kept at 100% from 13–18 min. From 18–26 min, buffer-B was linearly decreased from 100 to 75% and buffer-C was linearly increased from 0 to 25%. Buffer-C was linearly increased to 100% during 26–56 min and kept at 100% during 56–67 min. The buffer was changed to buffer-D and the proportion of buffer-D was kept at 100% during 67–74 min. The buffer was changed to buffer-A and the proportion of buffer-A was kept at 100% during 74–105 min. Detection of Phe was carried out using the same method as that for PEA. A standard sample was always analyzed within each batch to quantify aromatic amines and aromatic amino acids in the sample. Aromatic amine and aromatic amino acid species in the samples were identified by comparison with the retention times of the standards, and each compound concentration was determined using a standard curve generated from the peak areas of the standards at known concentrations.

### *Purification of unidentified biogenic amine from culture supernatant of* T. nexilis

The unidentified biogenic amine was purified from culture supernatant of *T. nexilis* using a cation exchange resin (Dowex 50 W X8, [H^+^-form; 100–200 mm], FUJIFILM Wako Pure Chemicals). Ten milliliters of resin was packed into a column. *T. nexilis* was anaerobically pre-cultured overnight in 1 mL of GAM at 37°C. Pre-cultures (500 μL) were inoculated into 500 mL of GAM and cultured overnight at 37°C in an anaerobic chamber (INVIVO2 400; Ruskinn Technology, Bridgend, UK) until stationary phase.^47^ The culture was centrifuged (12,680 *× g*, 4°C, 20 min), and the supernatant was applied to the resin-packed column. After loading, the unidentified biogenic amine was purified using the following procedures: the column was washed with 400 mL of deionized water. Polyamines (spermidine, cadaverine, and spermine) were eluted with 120 mL of 1 M HCl. Residual HCl was removed from the column by washing the column with 200 mL of deionized water. Agmatine was eluted with 115 mL of 500 mM of NH_3_ and the column was washed with 100 mL of deionized water. The unidentified biogenic amine was eluted with 100 mM HCl. The eluted unidentified amine was again absorbed onto the same column, washed with 2 L of deionized water, and then eluted by increasing the NH_3_ concentration in steps of 2, 4, and 8 M in 80 mL increments. Every 1 mL was recovered, and the concentration and purity of the unidentified biogenic amine were tested using HPLC. The fraction eluted with 8 M NH_3_ contained the highest amount of the unidentified biogenic amine, and was therefore subjected to further analysis.

### Determination of molecular mass of the purified unidentified biogenic amine using liquid chromatography coupled with tandem mass spectrometry (LC-MS/MS)

The mass of the purified product was determined using a 3200 QTRAP MS system (Applied Biosystems, Foster City, CA, USA). The sample was directly injected to the electrospray ionization-MS detector in scan mode with positive ionization. The molecular ion peak with *m/z* of 122 was further analyzed by MS/MS fragmentation. The purified sample was separated using an Agilent HPLC system (Agilent) as follows: column, TSKgel ODS-80Ts (4.6 × 250 mm^2^, 5 μm particles; Tosoh); solvent system, A: 0.1% acetic acid in water, B: 0.1% acetic acid in acetonitrile; gradient modes: 90% A (0–5 min), 90–60% A (5–20 min), and 10% A (20–30 min); flow rate, 0.5 mL/min at 40°C. The separated sample was analyzed by LC-MS/MS, using select ion mode at *m*/*z* = 122. Standard PEA was used for comparison. The biogenic amine was confirmed as PEA by comparing its retention time and daughter ions with that of standard PEA in LC-MS/MS analysis.

### Aromatic amine production profile of PEA-producing gut bacteria

PEA-producing gut bacteria were anaerobically pre-cultured in GAM at 37°C for 18 h in an anaerobic chamber (INVIVO2 400). After pre-cultivation, cells in the stationary phase^47^ were washed and resuspended with 30 mL of the AAAD medium (Supplementary Table S2) at an initial OD_600_ of 0.03 and cultured at 37°C in an anaerobic chamber. One milliliter of the culture was collected at the indicated times and centrifuged (18,900 *× g*, 4°C, 10 min) to obtain the culture supernatant. The culture supernatant was filtrated using a Cosmonice filter W (Nacalai Tesque) after protein precipitation by trichloroacetic acid, as described previously.^82^ The filtrated sample was analyzed using HPLC.

### *Aromatic amine production profile of* En. faecalis *and* R. gnavus *in the presence of human AADC inhibitors*

Human AADC inhibitors: carbidopa,^83^ methyldopa,^84^ and benserazide^85^ were dissolved in MilliQ water and added to the AAAD medium at a final concentration of 1.5 mM. The other culture conditions were the same as described above. Following 24 h cultivation in the presence of inhibitors, PEA and tyramine concentrations in the culture supernatants and OD_600_ were measured.

### Plasmid construction

Cells of *B. hansenii, C. asparagiforme, T. nexilis, En. faecalis*, and *R. gnavus* in 0.5 mL of overnight culture, were centrifuged and stored at −20°C until the genomic DNA (gDNA) extraction. Cells of *T. nexilis* and *R. gnavus* were suspended in 100 μL of TE buffer and disrupted using zirconia beads (Thermo Fisher Scientific, Waltham, MA, USA) in a SHAKE MASTER ver. 1.2 (Bio Medical Science, Tokyo, Japan). The disrupted cells were centrifuged (21,500 *× g*, 4°C, 10 min) and gDNA in the resulting supernatant fractions were used as templates in PCR reactions. *B. hansenii* and *C. asparagiforme* gDNA were extracted using the phenol-chloroform methods.^47^ *Enterococcus faecalis* gDNA was extracted using a Wizard Genomic DNA purification kit (Promega, Madison, WI), according to the manufacturer’s protocol. The genes of putative *aadc* (BLAHAN_06497 [*aadc*_Bh_], CLONEX_01451 [*aadc*_Tn_], and CLOSTASPAR_05940 [*aadc*_Ca_]) and *aadc* (EF_0634 [*aadc*_Ef_] and RUMGNA_01526 [*aadc*_Rg_]) were amplified using KOD-plus- Neo (Toyobo, Osaka, Japan) or PrimeSTAR Max (TaKaRa Bio, Shiga, Japan) from the respective gDNA using the primers listed in Table 1. Amplified DNA was cloned between the NdeI and XhoI sites of the expression vector, pCDF23^74^ using an In-Fusion HD cloning kit (Clontech Laboratories Inc., Mountain View, CA, USA). The *aadc* in the resulting plasmid was sequenced to ensure that there were no PCR-introduced errors. Plasmids were used to transform *Escherichia coli* BL21 (DE3) for heterologous expression.

Plasmids used for generating a standard curve of 16S rRNA genes and *aadc*_Ef_ in qPCR were constructed as follows: 16S rRNA gene and *aadc*_Ef_ were amplified from gDNA of *En. faecalis* using the primers listed in Table 1. Amplified DNA was cloned into SmaI site of pUC19 using DNA ligation kit Mighty Mix (TaKaRa Bio).

### *Heterologous expression of aadc in* Escherichia coli

*Escherichia coli* BL21 (DE3) strains harboring *aadc* expression plasmids or an empty plasmid were pre-cultured in 5 mL of Luria-Bertani medium containing 75 μg/mL spectinomycin in 100 mL Erlenmeyer flask, at 37°C with reciprocal shaking at 140 rpm for 17 h. M9 medium^86^ containing 0.2% of glucose and 1 mM of aromatic amino acids: Phe, Tyr, and Trp (M9AAA-medium) was used for the main culture. Pre-cultured cells were washed with M9 medium containing 0.2% glucose and suspended in M9AAA-medium. The suspension was inoculated into 10 mL of M9AAA-medium, supplemented with 10 μM isopropyl β-D-thiogalactopyranoside in a 100 mL Erlenmeyer flask at an initial OD_600_ of 0.03 and cultured at 37°C with reciprocal shaking at 140 rpm. An aliquot of the culture was collected every 6 h and centrifuged for 10 min at 21,500 *× g* at 4°C. The resulting culture supernatant was treated with 10% (*w*/*v*) trichloroacetic acid, and was subjected to HPLC analysis, as previously reported.^87^

### *Generation of deletion mutant of* aadc *in* En. faecalis *V583*

*The aadc* gene of *En. faecalis* V583 was deleted using pLT06 as described previously.^53^ Primers and plasmids used for *aadc* deletion and complementation are listed in Table 1. Complementation plasmid pLZ12-*aadc*^+^ was constructed as follows: *aadc* and its upstream 500 bp were amplified using PCR with PrimeSTAR Max DNA polymerase (TaKaRa Bio) with C_ddc+0.5K_F_pLZ_Bam and C_ddc+0.5K_R_pLZ_Bam as primers. The product was cloned into BamHI site of pLZ12. The resulting plasmid was introduced into *En. faecalis* by electroporation, as described previously.^53^

### Conversion of phenylalanine to phenylethylamine by human fecal sample

Fecal samples from nine healthy Japanese donors (eight male and one female; age: 21.8 ± 10.2, range 4–40 years) were analyzed. Feces were collected using stool collecting kit (LSI medience Co., Tokyo, Japan) and stored under anaerobic condition using Anaero Pack system (Mitsubishi Gas Chemical Co., Inc., Tokyo, Japan) at −80°C until use. Fecal samples were suspended in a 4-fold volume of phosphate-buffered saline (PBS) or PBS containing 1 mM of Phe and were incubated anaerobically at 37°C. After 8 h incubation, the culture supernatants were harvested by centrifugation (21,500 *× g*, 4°C, 10 min) and subjected to HPLC analysis, as described in the “*Aromatic amine producing profile of PEA-producing gut bacteria*” section. The net PEA production was calculated by subtracting PEA amount formed in the absence of Phe from that formed in the presence of 1 mM Phe in the fecal suspension.

### Extraction of gDNA from fecal samples

Fecal samples (10–20 mg) were suspended in 95 μL of TE buffer. Five microliters of 300 mg/mL lysozyme (Sigma-aldrich, MO. USA) and 11 μL of 1,000 U/μL achromopeptidase (FUJIFILM Wako Pure Chemicals) were added to the suspension and incubated for 30 min at 37°C. Twelve microliters of 20% sodium dodecyl sulfate solution was added to the suspension and incubated at 60°C for 20 min.^88^ The bacterial DNA was extracted from the incubated mixture using QIAamp Fast DNA Stool Mini Kit (QIAGEN, Hilden, Germany).

### Quantification of DNA copy numbers in fecal sample

The copy number of the targeted gene was determined by quantitative PCR (qPCR) using a thermal cycler (StepOne Real-time PCR system, Applied Biosystems) and TB Green *Premix Ex Taq* II (Tli RNaseH Plus) (TaKaRa Bio). Primers used for qPCR are listed in Table 1. The Reaction mixture (20 μL) consisted of 10 μL of 2× TB Green II mix, 9.2 ng of gDNA, and 0.7 μM (for *aadc* gene) or 0.35 μM (for 16S rDNA) of primers. PCR cycling conditions for the amplification of *aadc_Tn_* were 95°C for 30s, followed by 40 cycles of 95°C for 5 s and 64°C for 45s. PCR cycling conditions for the amplification of the other genes were 95°C for 30s, followed by 40 cycles of 95°C for 5 s and 60°C for 1 min. Gene copy numbers were calculated based on the standard curve generated using varying concentrations of gDNA or plasmids containing the target gene.

### Animal experiments

Six-week-old female BALB/cCrSlc mice were purchased from Japan SLC (Hamamatsu, Japan). All mice were maintained in a 12 h light-dark cycle and housed in group cages with 2‒3 animals per cage with free access to water and diet and bred based on the regulations regarding the protection of laboratory animals at the Kanazawa University. All animal experiments were performed according to the Guideline for the Care and Use of Laboratory Animals at Kanazawa University (Approval number: AP-163778). Mice were fed a standard diet (CE-2, CLEA Japan Inc., Tokyo, Japan) for 13 days. Tyr was removed from the diet and Phe content was increased from 0.87% (*w*/*w*) in the L-Amino Acid Defined AIN-93 G (Dyets Inc., Bethlehem, PA, USA) (Supplementary Table S5) to 8.7% (*w*/*w*) in the Phe-rich diet (#511379, Dyets Inc.) (Supplementary Table S5). The mice were fed a Phe-rich diet from for 5 d, beginning on day 13 (Figure 4b). Drinking water and the diet were sterilized by autoclave or γ-irradiation, respectively. Antibiotics (0.25 mg/mL of doripenem and 0.5 mg/mL of vancomycin) were provided ad libitum in the drinking water^89^ for 14 days until one day before *En. faecalis* administration.

The *En. faecalis* strains, WT, Δ*aadc*, and *aadc* complemented *En. faecalis* were grown anaerobically in GAM containing 10 μg/mL chloramphenicol at 37°C for 12 h. Cells were collected from 1 mL of culture by centrifugation (6,000 *× g*, 25°C, 5 min) and washed twice with 1 mL of PBS containing 10 μg/mL chloramphenicol. Cells were suspended in 5 mL of PBS and the colony forming unit (cfu) was measured on GAM agar plate containing 10 μg/mL chloramphenicol. Cells were prepared to 1 × 10^8^ cfu/200 μL and administered to mice. Three days after *En. faecalis* administration, serotonin content in the colon tissue was evaluated. These experiments were independently performed twice (n = 4 and 6 in each group, respectively), with a resulting n = 10 per group.

### Quantification of serotonin concentration in the colon tissue

Mice were euthanized by cervical dislocation under the anesthetization with Dormicum (Meiji Seika Pharma Co., Ltd., Tokyo, Japan), Vetorphale (Astellas Pharma, Inc., Tokyo, Japan), and Domitor (Nippon Zenyaku Kogyo Co., Ltd., Fukushima, Japan). The entire length of the colon with full-thickness was washed with PBS to remove the luminal contents, sonicated in 10 mL of PBS using Branson model 250 (BRANSON, St. Louis, MO, USA), and stored at −25°C, until use. The colon lysate was centrifuged (21,500 *× g*, 4°C, 5 min) to remove tissue debris. Serotonin concentration was determined using Serotonin ELISA Kit (Enzo Lifescience, Farmingdale, NY, USA), according to the manufacturer’s protocol. The serotonin ELISA Kit (Enzo Lifescience, Farmingdale, NY, USA) was used because the Serotonin ELISA Kit (Eagle Biosciences) used by Yano et al.^24^ could not be purchased in Japan due to legal restrictions. Serotonin amounts were normalized based on the weight of the colonic tissue. Animal experiments were independently performed twice (n = 4 and 6 in each group, respectively), and for each experiment, the amount of serotonin was normalized by the average amount of serotonin in mice colonized with wild-type *En. faecalis*. The normalized values were assigned an arbitrary unit (a.u.) and the serotonin levels in the colonic tissue were expressed as serotonin (a.u.).

### *Analysis of* Taar1 *and* Mao-b *expression in mouse colon*

Colon tissue from six-week-old female BALB/cCrSlc mice was purchased from Japan SLC. The tissue was treated with RNAlater-ICE (Thermo Fisher Scientific) for 20 h at −20°C and used for RNA extraction. The frozen colon was placed into a tube with a stainless bead and disrupted using a Multi-Beads shocker (Yasui Kikai Co., Osaka, Japan). RNA extraction and on-column genomic DNA digestion were then performed using ISOSPIN Cell & Tissue RNA Kit (Nippon Gene, Tokyo, Japan) according to the manufacturer’s instructions. Complementary DNA (cDNA) was synthesized from 0.2 or 1 μg of RNA using PrimeScript™ RT reagent Kit (Perfect Real Time) (TaKaRa Bio). TB Green® Premix Ex Taq™ (Tli RNaseH Plus) (TaKaRa Bio) was used for PCR, which was performed using 1 µL cDNA solution for samples that used 0.2 μg of RNA for cDNA synthesis and 0.2 μL cDNA solution for samples that used 1 μg of RNA for cDNA synthesis as a template. The PCR cycling conditions were 95°C for 30 sec, followed by 40 cycles of 95°C for 5 sec and 60°C for 30 sec. The PCR products were electrophoresed with 3% agarose gel and visualized using ethidium bromide under ultraviolet light.

### Statistical analysis

Statistical analyses were performed using SPSS software version 21 (IBM, Armonk, NY) and BellCurve for Excel (Social Survey Research Information Co., LTD.). Correlation between PEA production and *aadc* copy number was analyzed by Spearman’s rank correlation test. Repeated measures one-way ANOVA was employed to evaluate the significance of change in aromatic amine concentration with cultivation time. The Tukey-Kramer and Dunnett’s tests were used for multiple comparisons of aromatic amine concentrations. *aadc*_Ef_ and 16S rRNA gene copy numbers in mouse feces and colonic serotonin level were statistically analyzed using Kruskal-Wallis test followed by Steel-Dwass test. A *p* < .05 was considered statistically significant.

### Ethical approval

This study was approved by the Ethics Committee of Ishikawa prefectural university (2016–2) and was performed in accordance with the Declaration of Helsinki. Informed consent was obtained from all donors or their parents.

## Supplementary Material

Supplemental Material

## Data Availability

All necessary data are included in the manuscript since no omics analysis or determination of new genomes has been undertaken in this study.

## Data and materials availability

All data needed to evaluate the conclusions in this work are present in the paper and/or the Supplementary Materials. Additional data related to this paper will provided by the authors upon reasonable request.
