# Supplementary material for: Gut bacterial aromatic amine production: aromatic amino acid decarboxylase and its effects on peripheral serotonin production
Source: Gut Microbes. 2022 Oct 10;14(1):2128605. doi: 10.1080/19490976.2022.2128605 (PMC9553188; doi:10.1080/19490976.2022.2128605)
Supplement: Supplemental Material [file KGMI_A_2128605_SM7945.pdf]

Supplementary Materials for

Gut bacterial aromatic amine production: aromatic amino acid decarboxylase and its effects on peripheral serotonin production

Yuta Sugiyama, Yumiko Mori, Misaki Nara, Yusuke Kotani, Emiko Nagai, Hiroki Kawada, Mayu Kitamura, Rika Hirano, Hiromi Shimokawa, Akira Nakagawa, Hiromichi Minami, Aina Gotoh, Mikiyasu Sakanaka, Noriho Iida, Takashi Koyanagi, Takane Katayama, Shigefumi Okamoto, Shin Kurihara*

*Corresponding author
E-mail: skurihara@waka.kindai.ac.jp

This PDF file includes:

Supplementary Figure S1
HPLC chromatograms of the culture supernatants of PEA-producing gut bacteria grown in gifu anaerobic medium (GAM).

Supplementary Figure S2
Growth of PEA-producing gut bacteria in aromatic-amino-acid-defined (AAAD) medium.

Supplementary Figure S3
Expression of *Taar1* and *Mao-b* in the BALB/cCrSlc mouse colon.

Supplementary Figure S4
Concentration of tyramine in mouse feces and correlation of colonic serotonin.

Supplementary Figure S5
OD_{600} and the concentrations of PEA and tyramine in the culture supernatants of *En. faecalis* and *R. gnvaus* cultivated for 24 h under the presence of human AADC inhibitor.

Supplementary Table S1
Amino acid composition of GAM (Lot: 115607).

Supplementary Table S2
Composition of aromatic-amino-acid-defined (AAAD) medium.

Supplementary Table S3
Statistical analysis of cultivation time-dependent aromatic amine production by PEA-producing gut bacteria.

Supplementary Table S4
Statistical analysis of cultivation time-dependent aromatic amine production by *E. coli* transformants.

Supplementary Table S5
Standard and phenylalanine rich diet composition.

References.

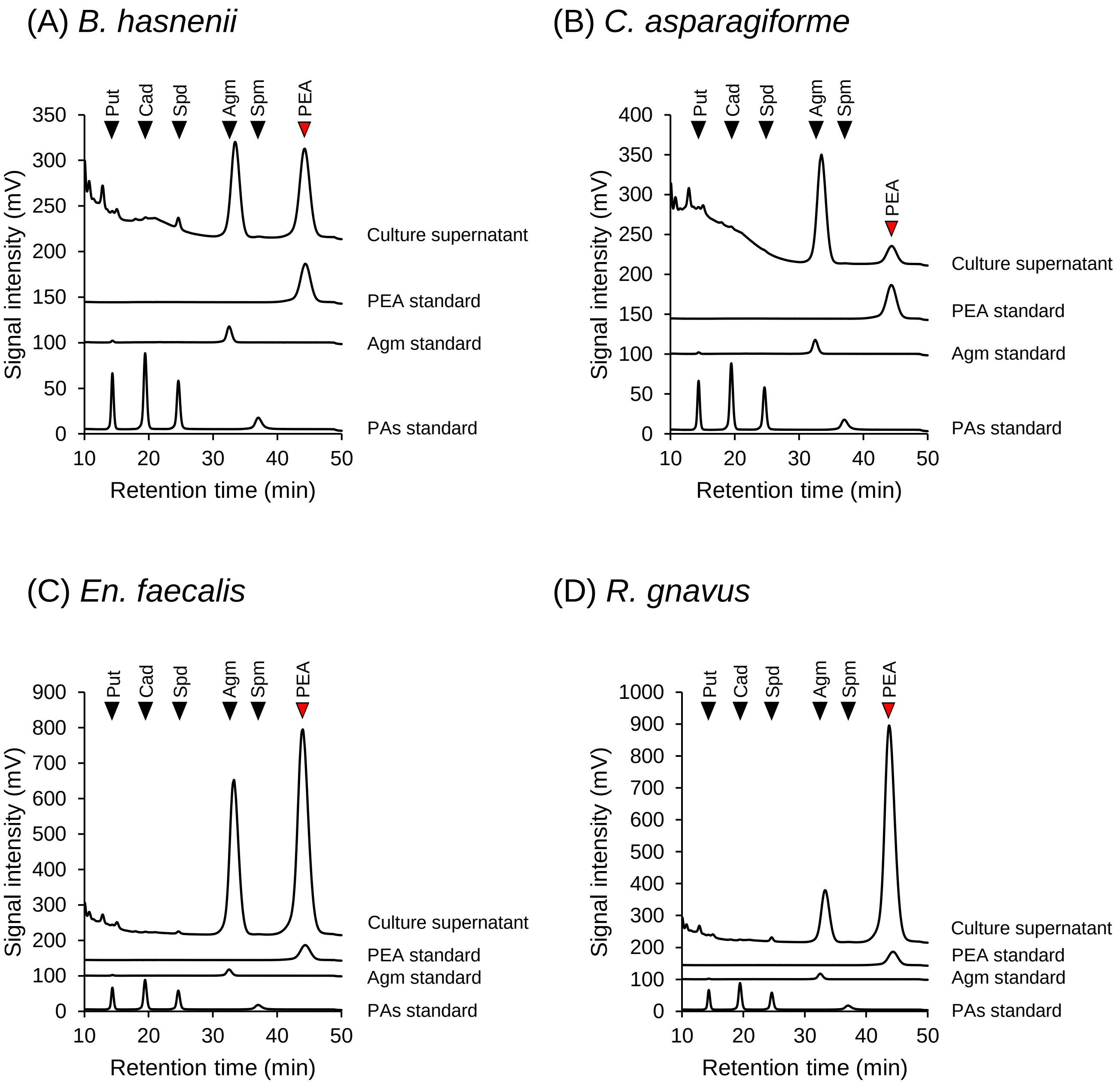

Supplementary Figure S1. HPLC chromatograms of the culture supernatants of PEA-producing gut bacteria grown in gifu anaerobic medium (GAM).

Blautia hansenii (A), *Clostridium asparagiforme* (B), *Enterococcus faecalis* (C), and *Ruminococcus gnavus* (D) were cultured for 30 h in the GAM, and the culture supernatants were analyzed. The chromatograms of polyamines (PAs) standard (Put, putrescine; Cad, cadaverine; Spd, spermidine; Spm, spermine), agmatine (Agm) standard, and PEA standard are also shown.

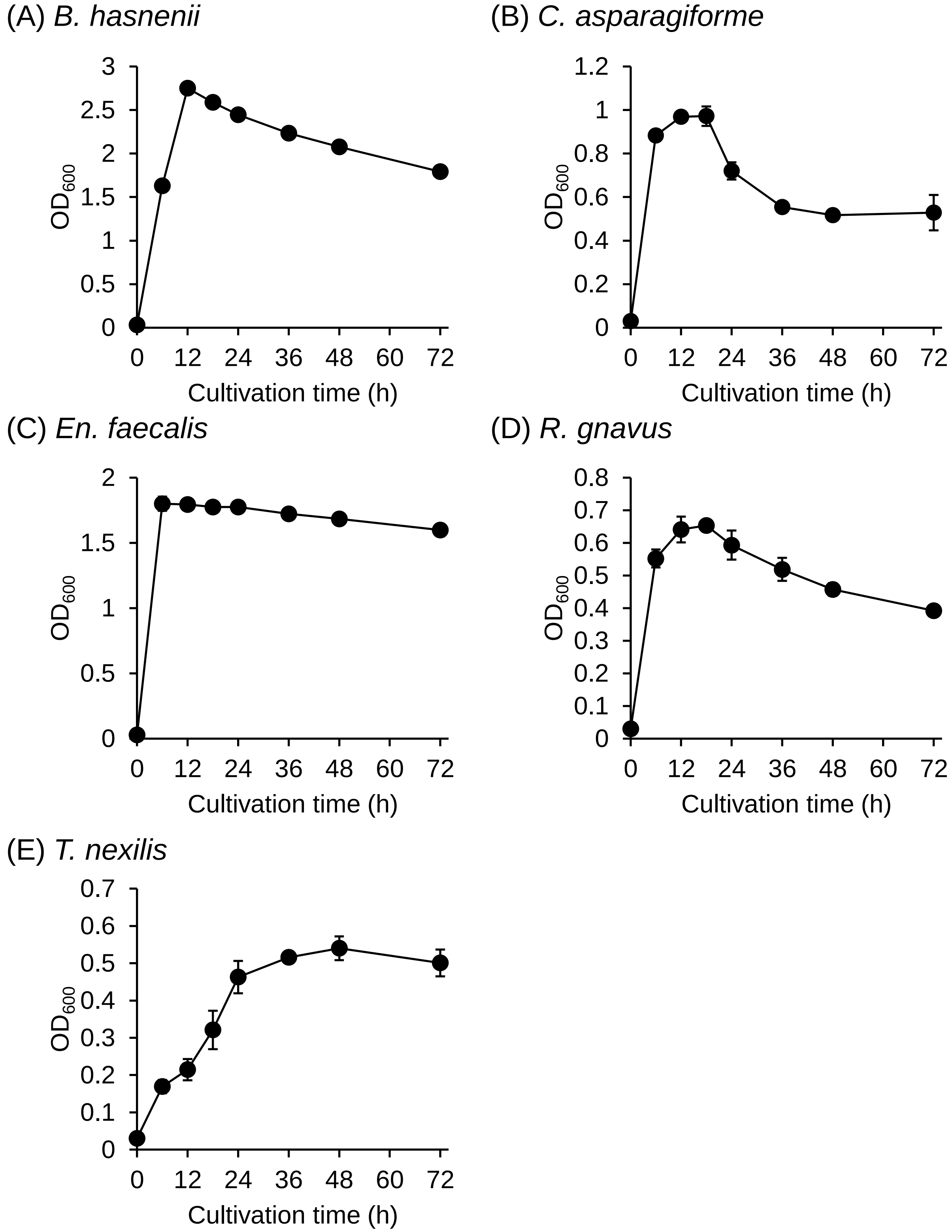

Supplementary Figure S2. Growth of PEA-producing gut bacteria in aromatic-amino-acid-defined (AAAD) medium.

(A) *Blautia hansenii*, (B) *Clostridium asparagiforme*, (C) *Enterococcus faecalis*, (D) *Ruminococcus gnavus*, and (E) *Tyzzerella nexilis* were grown in AAAD medium and OD_{600} values were measured at the indicated time. Data represent the mean ± SD of three individual experiments.

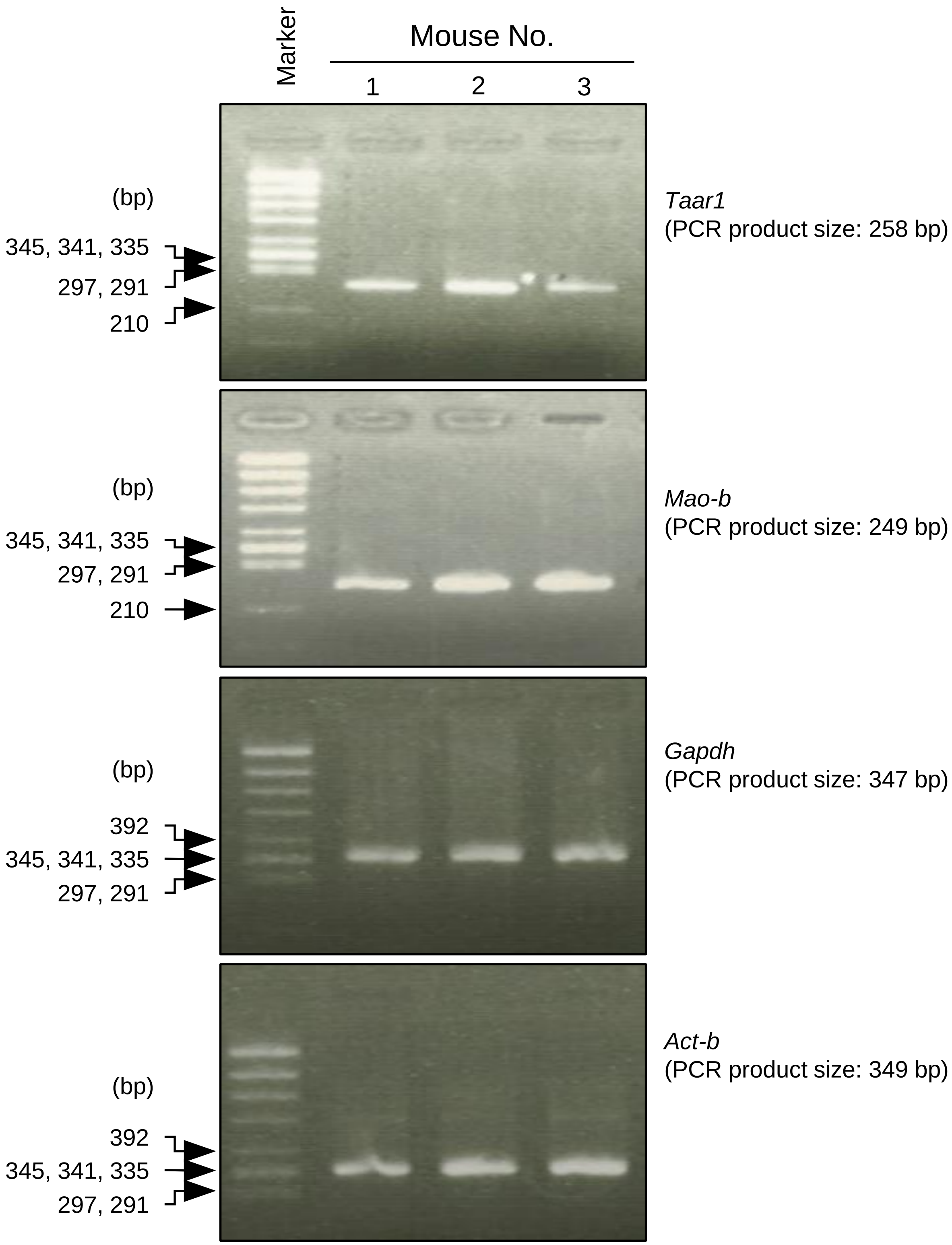

Supplementary Figure S3. Expression of *Taar1* and *Mao-b* in the BALB/cCrSlc mouse colon.

Taar1 and *Mao-b* gene expression were analyzed by reverse transcriptional PCR. Complementary DNA was synthesized from RNA purified from six-week-old female BALB/cCrSlc mouse colon and used as a PCR template. PCR products were visualized using ethidium bromide under UV light. OneSTEP marker 5 (Nippon Gene, Tokyo, Japan) was used as a size marker.

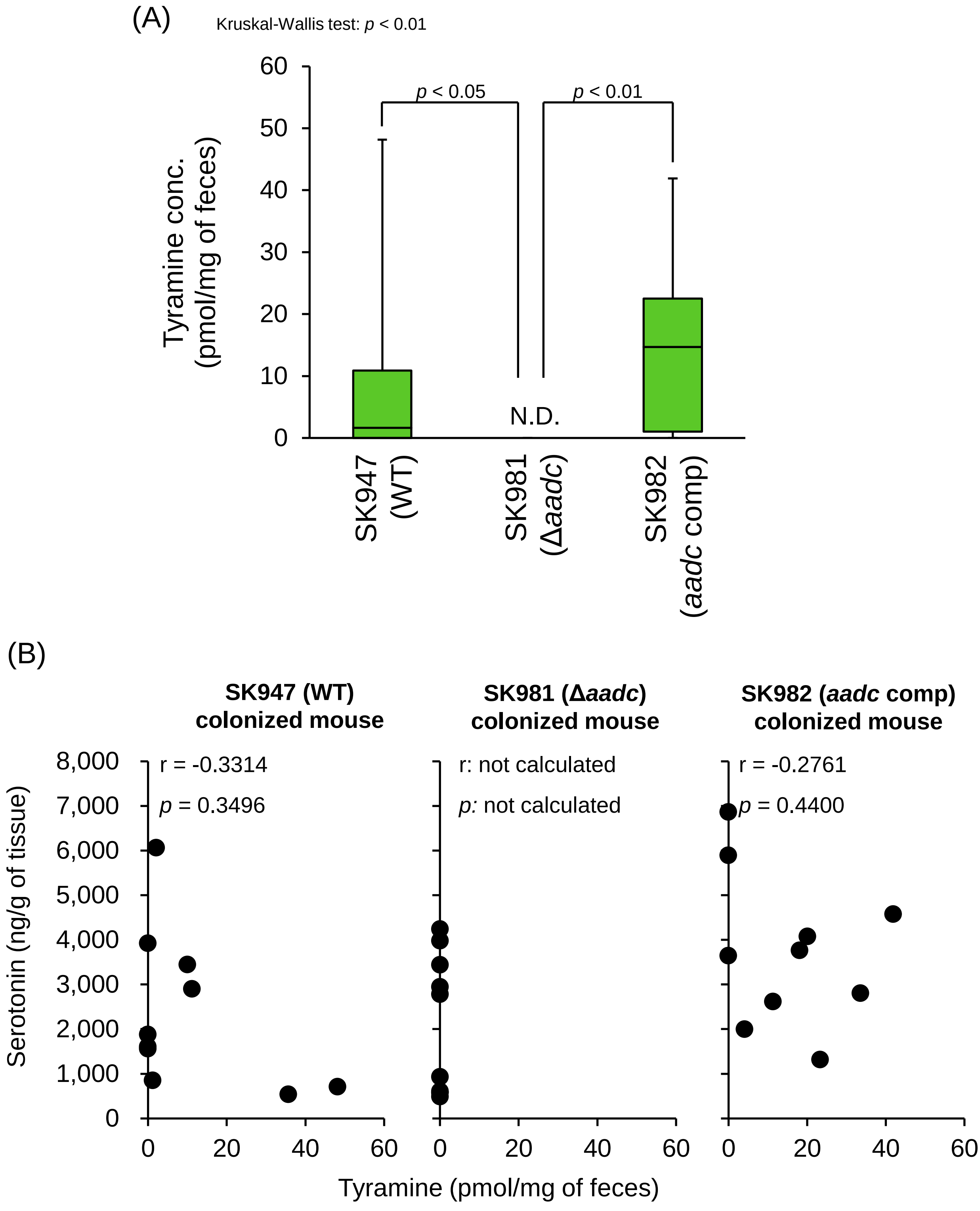

Supplementary Figure S4. Concentration of tyramine in mouse feces and correlation of colonic serotonin.

(A) Tyramine amounts in mouse feces.
The two separate experiments were performed (n = 4 and 6, respectively), and data are shown by box plots, in which the horizontal line inside the box is the median. Statistical analysis was performed using Kruskal-Wallis test followed by Steel-Dwass test. The *p*-values for Kruskal-Wallis test and Steel-Dwass test are shown. N.D., not detected.
(B) Correlation between Fecal tyramine amounts and colonic serotonin amounts.
Spearman's rank correlation test was used for the correlation analysis (r = correlation coefficient).

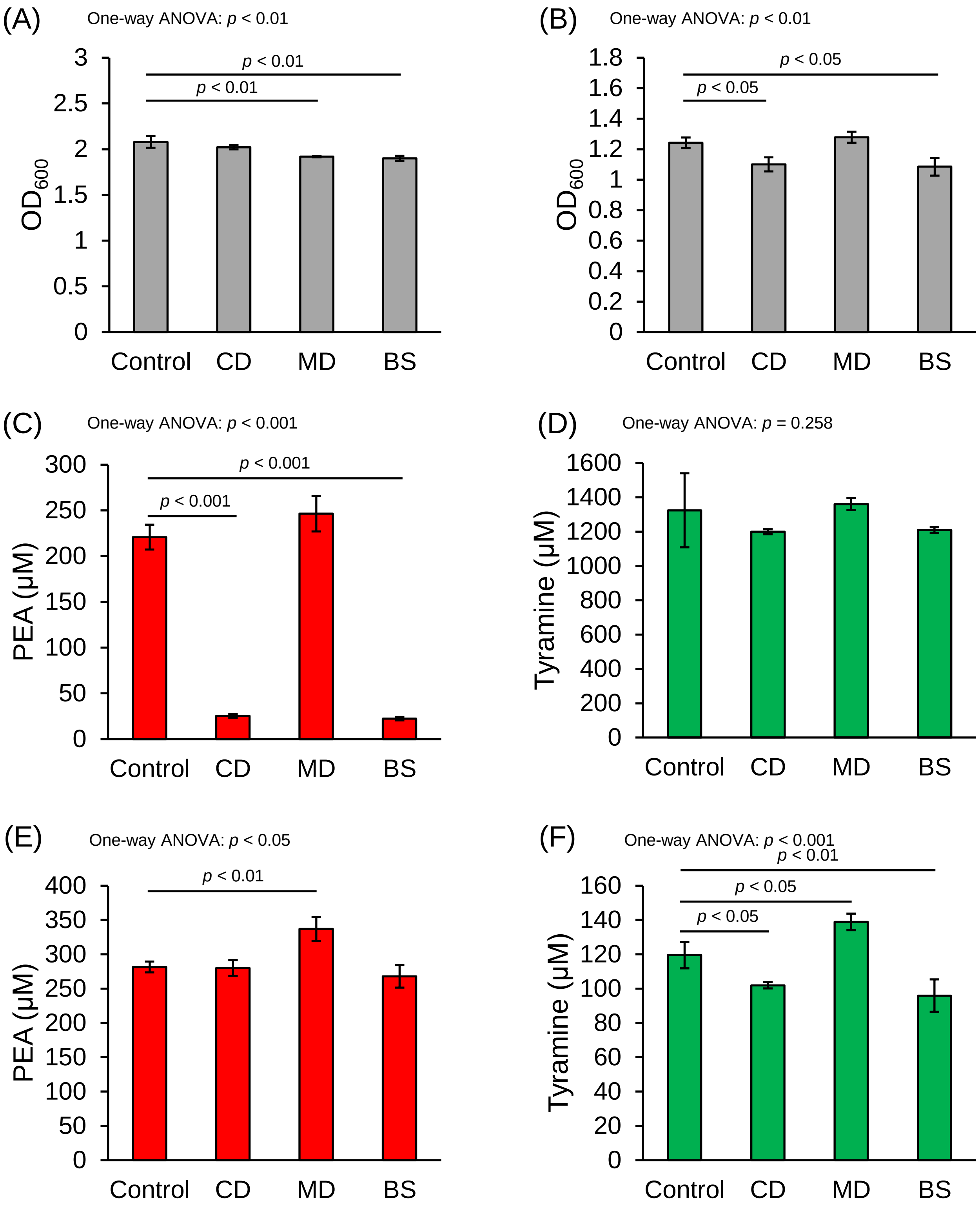

Supplementary Figure S5. OD_{600} and the concentrations of PEA and tyramine in the culture supernatants of *En. faecalis* and *R. gnvaus* cultivated for 24 h under the presence of human AADC inhibitor.

The abbreviations used are as follows: CD, Carbidopa; MD, methyldopa; BS, benserazide.
(A) OD_{600} of *En. faecalis*.
(B) OD_{600} of *R. gnavus*.
(C) PEA concentration in the culture supernatants of *En. faecalis.*
(D) Tyramine concentration in the culture supernatants of *En. faecalis*.
(E) PEA concentration in the culture supernatants of *R. gnavus*.
(F) Tyramine concentration in the culture supernatants of *R. gnavus*.
Data represent the mean ± SD of three independent experiments. Statistical analyses were performed by one-way ANOVA followed by Dunnett's test. The *p*-values for one-way ANOVA and Dunnett's test are shown.

Supplementary Table S1. Amino acid composition of GAM (Lot: 115607).

Amino acid	Concentrations (mM)[a]
Alanine	9.08
Arginine	4.98
Aspartic acid	3.60
Cysteine	0.10
Glutamic acid	6.79
Histidine	1.53
Isoleucine	4.03
Leucine	12.14
Lysine	7.54
Methionine	2.01
Phenylalanine	5.50
Proline	2.53
Serine	4.82
Threonine	4.30
Tryptophan	1.36
Tyrosine	0.89
Valine	6.40

[a]The concentrations in the medium, prepared according to the manufacture’s protocol.

Supplementary Table S2. Composition of aromatic-amino-acid-defined (AAAD) medium.

Reagents	Final concentrations
D-Glucose	0.5 % (*w*/*v*)
10× Bacteroides minimal salts[a]	1×
400 mM L-Cysteine·HCl·H_2O	4 mM
1.9 mM Hematin/200 mM L-Histidine	1.9 µM Hematin/200 µM L-Histidine
1 mg/mL Vitamin K_3	1 µg/mL
10 µg/mL Vitamin B_{12}	5 ng/mL
100 mM $MgCl_2 \cdot 6H_2O$	100 µM
1.4 mM $FeSO_4 \cdot 7H_2O$	1.4 µM
50 mM $CaCl_2 \cdot 2H_2O$	50 µM
10 mM L-Tryptophan	1 mM (including Trp contained in GAM)
2 mM L-Tyrosine	1 mM (including Tyr contained in GAM)
50 mM L-Phenylalanine	1 mM (including Phe contained in GAM)
GAM (Lot:115607)	10 %

[a]10× Bacteroides minimal salts containing 1 M KH_2PO_4, 150 mM NaCl, and 85 mM $(NH_4)_2SO_4$; pH adjusted to 7.2 using NaOH[1-2].

Supplementary Table S3. Statistical analysis of cultivation time-dependent aromatic amine production by PEA-producing gut bacteria.

Bacteria	Aromatic amine	Repeated measures one-way ANOVA		Tukey-Kramer test[a] (0 h vs 72 h)
B. hansenii	PEA	$F = 160$	$p < 0.001$	$p = 0.003$
	Tyramine	$F = 2$	$p = 0.311$	−
	Tryptamine	$F = 22$	$p = 0.041$	$p = 0.033$
C. asparagiforme	PEA	$F = 69$	$p = 0.010$	$p < 0.001$
	Tyramine	$F = 33$	$p = 0.016$	$p = 0.003$
	Tryptamine	$F = 4$	$p = 0.191$	−
En. faecalis	PEA	$F = 85$	$p = 0.003$	$p = 0.008$
	Tyramine	$F = 156$	$p < 0.001$	$p = 0.008$
	Tryptamine	$F = 1$	$p = 1.000$	−
R. gnavus	PEA	$F = 300$	$p < 0.001$	$p = 0.002$
	Tyramine	$F = 135$	$p = 0.002$	$p = 0.003$
	Tryptamine	$F = 161$	$p = 0.004$	$p = 0.005$
T. nexilis	PEA	$F = 108$	$p = 0.004$	$p < 0.001$
	Tyramine	$F = 10$	$p = 0.029$	$p = 0.003$
	Tryptamine	$F = 33$	$p = 0.017$	$p = 0.022$

[a]Tukey-Kramer test was performed only when repeated measures one-way ANOVA results were significant ($p < 0.05$).

Supplementary Table S4. Statistical analysis of cultivation time-dependent aromatic amine production by *E. coli* transformants.

E. coli strain (harboring *aadc*)	Aromatic amine	Repeated measures one-way ANOVA		Tukey-kramer test[a] (0 h vs 48 h)
YS389 ($aadc_{Bh}$)	PEA	$F = 121$	$p = 0.007$	$p = 0.010$
	Tyramine	$F = 49$	$p = 0.016$	$p < 0.001$
	Tryptamine	$F = 257$	$p = 0.002$	$p = 0.003$
YS300 ($aadc_{Ca}$)	PEA	$F = 45$	$p = 0.008$	$p < 0.001$
	Tyramine	$F = 91$	$p = 0.003$	$p = 0.002$
	Tryptamine	$F = 31$	$p = 0.014$	$p < 0.001$
YS300 ($aadc_{Ef}$)	PEA	$F = 240$	$p = 0.002$	$p = 0.001$
	Tyramine	$F = 118$	$p = 0.005$	$p < 0.001$
	Tryptamine	–[b]	–[b]	–[b]
YS300 ($aadc_{Rg}$)	PEA	$F = 270$	$p < 0.001$	$p < 0.001$
	Tyramine	$F = 57$	$p = 0.004$	$p = 0.004$
	Tryptamine	$F = 228$	$p < 0.001$	$p < 0.001$
YS300 ($aadc_{Tn}$)	PEA	$F = 715$	$p < 0.001$	$p < 0.001$
	Tyramine	$F = 152$	$p < 0.001$	$p = 0.003$
	Tryptamine	$F = 107$	$p = 0.006$	$p = 0.002$

[a]Tukey-Kramer test was performed only when repeated measures one-way ANOVA results were significant ($p < 0.05$).

[b]Tryptamine production by YS300 was not detected in our HPLC system.

Supplementary Table S5. Standard and phenylalanine rich diet[a] composition.

Components	L-Amino Acid Defined AIN-93G	Phe-rich
	g/kg	g/kg
L-Alanine	4.5	3.7
L-Arginine (free base)	6.3	5.13
L-Aspartic acid	11.3	9.22
L-Cysteine	3.7	3
L-Glutamic acid	36.2	10.29
Glycine	3.1	2.52
L-Histidine (free base)	4.5	3.66
L-Isoleucine	8.4	6.84
L-Leucine	15.3	12.36
L-Lysine·HCl	16.1	13.05
L-Methionine	4.5	3.62
L-Phenylalanine	8.7	87
L-Proline	20.4	16.52
L-Serine	9.4	7.58
L-Threonine	6.6	5.36
L-Tryptophan	2.1	1.75
L-Tyrosine	9.2	0
L-Valine	9.9	8.03
Sucrose	100	100
Corn starch	399.886	380.46
Dyetrose	145	145
Soybean oil	70	70
tBHQ[b]	0.0014	0.014
Cellulose	50	50
Sodium bicarbonate	7.4	7.4
Vitamin mix #310025	10	10
Salt mix #210030	35	35
Choline bitartrate	2.5	2.5

[a]Phe- rich diet was prepared from L-Amino Acid Defined AIN-93G diet.

[b]*tert*- Butylhydroquinone.

References

1. Koropatkin, N.M., Martens, E.C., Gordon, J.I., and Smith, T.J. Starch catabolism by a prominent human gut symbiont is directed by the recognition of amylose helices. *Structure.* **16**, 1105-1115 (2008).

2. Sakanaka, M., Sugiyama, Y., Kitakata, A., Katayama, T., and Kurihara, S. Carboxyspermidine decarboxylase of the prominent intestinal microbiota species *Bacteroides thetaiotaomicron* is required for spermidine biosynthesis and contributes to normal growth. *Amino Acids.* **48**, 2443-2451 (2016).
